# Prevalence and Impact of Single-Day Events of Sexual Harassment, Racial Mistreatment, and Incivility on Biomedical Health Trainees: A Mixed-Methods Study

**DOI:** 10.3390/bs16030380

**Published:** 2026-03-06

**Authors:** Margaret S. Stockdale, Ann C. Kimble-Hill, Amanda E. Mosier, Jessica Kiebler, Breianna R. N. Mildor, Darius M. Washington

**Affiliations:** 1 Department of Psychology, Indiana University, Indianapolis 402 N. Blackford St., Indianapolis, IN 46202, USA; amandamosier94@gmail.com (A.E.M.); jkiebler@iu.edu (J.K.); mildorbr@mail.uc.edu (B.R.N.M.); dariwash@iu.edu (D.M.W.); 2 Indiana University Simon Comprehensive Cancer Center, Indianapolis, IN 46202, USA; ankimble@iu.edu; 3 Zurich North America, Schaumburg, IL 60196, USA; 4 Sojourner House, Providence, RI 02908, USA; 5 Department of Psychology, University of Cincinnati, Cincinnati, OH 45219, USA

**Keywords:** sexual harassment, racial harassment, microaggressions, incivility, positive and negative affect, attitudes, survey, qualitative data, biomedical health trainees

## Abstract

Little research has examined how often biomedical trainees encounter mistreatment in a single day or how such momentary experiences may undermine engagement in training. To address this gap, we investigated the prevalence and short-term consequences of daily sexual harassment, racial mistreatment, and incivility among graduate students and post-doctoral fellows in U.S. biomedical programs. In Study 1, 404 National Institutes of Health-funded trainees completed a two-wave survey assessing mistreatment, mood, and program attitudes across two 24 h periods separated by 10 days. On either day, 36.9% of participants experienced or observed at least one mistreatment episode, with no differences by gender or underrepresented minority status. Day 1 mistreatment was significantly negatively associated with program attitudes 10 days later, suggesting short-term derailment effects. In Study 2, 21 participants responded to true accounts of peers’ mistreatment to describe their emotional reactions and expectations of mentors. Trainees reported anger, disgust, and betrayal, and emphasized the need for mentors to acknowledge these harms, intervene appropriately, and offer support. This study provides the first evidence of single-day mistreatment prevalence among biomedical health trainees and demonstrates that even brief exposures can degrade training program attitudes. Findings underscore the need for improved mentor training and institutional resources to protect and support trainees.

## 1. Introduction

The mistreatment of students in higher education has been well documented. In a study of over 180,000 undergraduate and graduate students from 33 U.S. universities, the Association of American Universities (AAU) found that nearly 42% reported experiencing a sexually harassing behavior at least once since they were enrolled in college (Cantor et al., 2020). Racial microaggressions are so ubiquitous among Black, Latino/a, Asian, and other minoritized students that prevalence estimates are meaningless (Nadal et al., 2014; Cénat et al., 2022). Incivility, bullying, and other types of mistreatments of trainees are also commonplace (Abate & Greenberg, 2023; Mgboji et al., 2021; Moss & Mahmoudi, 2021; Pattani et al., 2018; Roberts, 2023). The impact of these experiences is not trivial. The AAU study found that over 45% of harassed students reported interference with academic or professional performance, limiting their ability to participate in an academic environment, or experiencing a hostile, intimidating, offensive environment (Cantor et al., 2020). The mistreatment of research trainees poses a significant threat to the scientific and biomedical health workforce with downstream consequences to scientific discovery and population health due to the derailing consequences of abuse during this important developmental career stage.

Despite the well-publicized documentation of these abuses, there appears to be no let-up. The National Institutes of Health (NIH) in the U.S., for example, has been tracking and responding to incidents of harassment and other forms of professional mistreatment among funded researchers since 2019 and have found that reports of harassment have outpaced research misconduct and foreign interference as the number one reported offense (Lauer, 2021; Working Group Report to the Advisory Committee to the NIH Director [ACD], 2019). To raise the alarm higher, we report findings from a national study of graduate students and post-doctoral fellows in biomedical health training programs in the U.S. who were surveyed on their single-day experiences of sexual harassment, racial harassment, racial microaggressions, and incivility in order to document the breadth and inter-connectedness of those experiences and their impacts on trainees’ retention-focused attitudes. We also gathered trainees’ reactions to real-life stories about these experiences and their insights on how they would have wanted such experiences to be handled by their mentors and faculty leaders. Together, our research not only documents the prevalence and inter-connections between multiple forms of mistreatment that occur daily but also provides compelling pleas from trainees for their leaders and mentors to pay attention and stop the abuse. After briefly reviewing the literature on these multiple forms of mistreatment in academic training contexts, we present our survey findings and the results of our qualitative analysis of trainees’ reactions to first-person accounts of abuse in these settings.

This study makes three contributions. First, it shifts the unit of analysis from estimating the prevalence of these forms of mistreatment across either vague (e.g., lifetime) or long intervals (e.g., past two years) to discrete, short-term mistreatment events. Second, it demonstrates that even isolated events are associated with meaningful changes in retention-relevant attitudes and affect. Third, by integrating trainees’ perspectives on stories of mistreatment, the study clarifies how trainees interpret and contextualize these events, informing intervention design.

### 1.1. Literature Review

The bodies of literature on interpersonal mistreatment have largely been segmented by the type of mistreatment: sexual harassment, racial harassment, racial microaggression, and incivility. Although our review is not exhaustive, we highlight representative research documenting the incidence, context, interconnects, and impacts of these forms of mistreatment of students in academic training, highlighting the biomedical research training context, including science, technology, engineering, mathematics (STEM), health, and medicine programs.

#### 1.1.1. Sexual Harassment

Although sexual harassment has a legal definition developed through case law in the U.S. (e.g., *Faragher v. City of Boca Raton*, 1998) and one promulgated by the U.S. Equal Employment Opportunity Commission (E.E.O.C., 1997), scholars typically rely on the psychological definition because it captures categories of sex-related behavior that are experienced as offensive or threatening regardless of whether it rises to a legal standard of sexual harassment, particularly regarding frequency and severity (Fitzgerald et al., 1995b, 1997). In fact, a purpose of this paper is to demonstrate that single-day episodes of sexual harassment have adverse effects. Fitzgerald and colleagues developed definitions of sexual harassment around three categories of behaviors: (a) sexual coercion (sexual advances that condition terms of employment or education upon sexual cooperation); (b) unwanted sexual attention (verbal and nonverbal behaviors that is offensive, unwanted, and unreciprocated); and (c) gender harassment (verbal and nonverbal behavior “not aimed as at sexual cooperation but that convey insulting, hostile, and degrading attitudes about women” (Fitzgerald et al., 1995a, p. 430). Gender harassment breaks into two forms: sexist hostility (demeaning jokes and comments about one’s gender) and sexual or crude hostility (use of sexually crude, degrading terms based on gender; Fitzgerald et al., 1995b; Stark et al., 2002).

Ranging from undergraduate students, graduate and professional students to medical residents, recent research finds astonishing rates of sexual harassment experience in STEM and other biomedical health educational training contexts. Aycock et al. (2019) surveyed 471 female undergraduates attending the 2017 American Physical Society’s Undergraduate Women in Physics conference. While in the context of physics education, a quarter reported experiencing gender harassment consisting of sexualized hostility and sexist hostility and a fifth reported those experiences as well as unwanted sexual attention. In a survey that included students and post-doctoral fellows in biological anthropology (total N = 66), nearly 65% reported experiencing some form of sexual harassment, with women being 3.5 times more likely than men to experience sexual harassment or sexual assault (Clancy et al., 2014). A study of 327 Canadian medical residents found 807 incidents of harassment, the most common being inappropriate remarks (29% of incidents), suggestive looks (22% of incidents), and suggestive physical gestures (11% of incidents; Phillips et al., 2019). A survey of 204 podiatry residents in the US reported that 82% of women and 42% of men had experienced sexual hostility, 26% and 7%, respectively, experienced unwanted sexual attention, and 8% and 4%, respectively, experienced sexual coercion during their residencies (Ang & Schneider, 2021). Clancy et al.’s (2017) survey of planetary science members that included undergraduates through tenured professors also found high rates of experiences of sexist remarks (79% of women and 63% of men) and harassment for not being masculine or feminine enough (60% of women and 33% of men) among other forms of harassment (Clancy et al., 2017).

#### 1.1.2. Racial Mistreatment

Mistreatment of racial minorities includes not only experiences common to non-minorities but also those that are rooted in racist stereotypes and racial animus, including racialized sexual harassment, racial microaggressions, and race discrimination. Racialized sexual harassment is an intersection of sexual and racial harassment, defined as “race-based differential treatment that may create a pervasive hostile environment” (Buchanan & Fitzgerald, 2008, p. 138), that people (typically women) of color experience as a function of their gender and race (Buchanan & Ormerod, 2002; Settles, 2006). It is often fueled by stereotypes of racial minorities, such as the “Jezebel” and “Sapphire,” which convey stereotypes of overly sexualized and angry Black women (Anderson et al., 2018; Buchanan & Ormerod, 2002). Extant research does not provide a clear picture of the prevalence of experiencing racialized sexual harassment in training contexts, but voices of minority women indicate that these experiences are common, rooted in racist and harmful stereotypes of women of color (Buchanan et al., 2018; Buchanan & Ormerod, 2002; Settles, 2006; Szymanski & Lewis, 2016). In a study of 2009 undergraduate students on their experiences of both sexual and racial harassment, Buchanan et al. (2009) found that Black, Asian, and multicultural students were significantly more likely than White students to experience racial harassment and that Asian and multiracial students were more likely to experience sexual harassment than White students. These experiences were associated with a multitude of mental health outcomes, which tended to be stronger for students of color (Buchanan et al., 2009). In a study of 212 Black female students, Szymanski and Lewis reported significant correlations of racialized sexual harassment experiences (which they labeled as “gendered racism”) with psychological distress, which was especially strong among those who highly identified as a Black woman (Szymanski & Lewis, 2016). A study of 205 graduate students in top political sciences programs in the US found that 40% of men of color and 48% of women of color experienced racialized harassment, which correlated negatively with graduate student satisfaction—a sign of derailment.

In addition to racial harassment and racialized sexual harassment, racialized microaggressions, defined as “brief and commonplace daily verbal, behavioral, and environment indignities, whether intentional or unintentional, that communicate hostile, derogatory, or negative racial slights and insults toward people of color” (Sue et al., 2007, p. 271), are also common and harmful in academic training contexts. Racial microaggression derails students of color by interfering with their learning and by wearing them down from the constant stress of experiencing and confronting these subtle but persistent mistreatments (Ogunyemi et al., 2020). A qualitative study of 24 students of color in STEM fields revealed themes of isolation and a culture of competition in their research labs. They reported that mentors preferentially treated star students or were effectively absent from the lab allowing micro-abuses to go unaddressed (Rodriguez et al., 2022). In a sample of 105 Black freshmen, Le et al. found that over 95% experienced a form of “everyday racism” (i.e., microaggression) in the past year, with over two-thirds experiencing such treatment multiple times (Le et al., 2021). In Clancy et al.’s (2017) study of learners and educators in planetary sciences described above, people of color, more so than Whites, reported racist remarks from peers (58% vs. 43%, respectively) and others (68% vs. 54%). These experiences predicted skipping school and work events—again, signs of derailment.

#### 1.1.3. Incivility

Incivility is defined as “low-intensity deviant behavior with ambiguous intent to harm the target, in violation of workplace norms for mutual respect” (Andersson & Pearson, 1999, p. 457). Incivility behaviors are characteristically rude and discourteous, displaying a lack of regard for others (Andersson & Pearson, 1999). The study of incivility has extended to academic training contexts by multiple researchers (Grover et al., 2020; Mgboji et al., 2021; Moss & Mahmoudi, 2021; Pattani et al., 2018; Rieger et al., 2023), and its occurrence correlates with other forms of mistreatment, including sexual harassment (Abate & Greenberg, 2023; Clancy et al., 2017; Lim & Cortina, 2005) and racial microaggressions (Clancy et al., 2017). Incivility behaviors and related conduct, such as bullying, in academic training contexts include ridiculing, public blaming and shaming, invasion of privacy, public put-downs and humiliation, interference with matriculation, removing funding, writing falsely negative letters of recommendation, taking credit for others work, threatening to cancel visas and fellowships, eye rolling, gossip, derogatory comments, social exclusion, blatant interruptions, talking over, throwing objects, toxic online behaviors, dismissing others’ scientific work; the list goes on (Abate & Greenberg, 2023; Mgboji et al., 2021; Moss & Mahmoudi, 2021; Pattani et al., 2018; Rieger et al., 2023; Roberts, 2023). The Association of American Medical Colleges’ 2022 Medical School Graduate Questionnaire, completed by almost 16,000 first-year medical residents found that 20% had experienced some form of public humiliation (Roberts, 2023), and a review of research articles reporting on the incidences of incivility (and bullying), sexual harassment, and racial harassment across the spectrum of medical training concluded that among medical students 17% experienced incivility and up to 60% experienced harassment and discrimination. In many reports, women and students of color were found to experience incivility at higher rates compared to men and Whites (Abate & Greenberg, 2023; Keller et al., 2020; Mgboji et al., 2021; Moss & Mahmoudi, 2021; Roberts, 2023). Perpetrators of incivility tended to be in positions of greater power, such as principal investigators, professors, and medical residents (toward medical students) (Abate & Greenberg, 2023; Grover et al., 2020; Moss & Mahmoudi, 2021). Experiencing incivility, like the other forms of mistreatment reviewed here, is associated with harmful outcomes, including isolation, burnout, and depression, feelings of worthlessness and guilt, physiological somatic complaints, changing career specialties, and even patient safety (Abate & Greenberg, 2023; Pattani et al., 2018; Roberts, 2023).

#### 1.1.4. Ambient Mistreatment

Research on ambient and bystander harassment provides a strong theoretical foundation for distinguishing among individuals who directly experience mistreatment, those who observe it, and those who do both. In their integrated model of ambient sexual harassment, Glomb et al. (1997), conceptualized indirect exposure as a job stressor that predicts dissatisfaction and psychological distress even among employees who are not direct targets. Extending this framework to race, Chrobot-Mason et al. (2013) situate ambient racial harassment within Conservation of Resources (COR) theory (Hobfoll, 1989) and stress-and-racism models (Brondolo et al., 2009; Clark et al., 1999), arguing that awareness of racial harassment depletes psychological resources and functions as a unique workplace stressor. Their findings indicate that awareness of biased behaviors and offensive racial comments is associated with lower job satisfaction, greater turnover intentions, and heightened psychological strain, underscoring the affective and attitudinal consequences of indirect exposure. Similarly, observing incivility is associated with heightened anger, fear, anxiety, and demoralization (Miner & Eischeid, 2012), suggesting that “bystander incivility” produces a stress response. Complementing these models, research on bystander sexual harassment similarly demonstrates that observing harassment independently predicts diminished well-being and withdrawal-related outcomes, even after accounting for direct victimization (e.g., Hitlan, 2026). Across these frameworks, both direct and indirect mistreatment are understood as stressors that consume emotional and cognitive resources, heighten vigilance, and shape perceptions of climate and safety. Accordingly, distinguishing among those who only experience mistreatment, only observe mistreatment, or both experience and observe mistreatment allows for a theoretically grounded examination of whether positive affect, negative affect, and program attitudes differ across these exposure types.

#### 1.1.5. Impacts of Mistreatment on Career Derailment

Meta-analytic and large-sample evidence consistently shows that both direct exposure to and (to a lesser extent) observation of sexual and racial harassment, racial microaggressions, and incivility are associated with a broad pattern of psychological distress (e.g., anxiety, depression, strain), negative affect (elevated negative affect and reduced well-being), and somatic complaints (e.g., physical symptoms and poorer health), alongside attitudes central to persistence versus derailment from career pursuits. Workplace sexual harassment is reliably linked to lower job satisfaction and organizational commitment, greater withdrawal and turnover intentions, and poorer psychological and physical health (Chan et al., 2008; Willness et al., 2007). Meta-analytic evidence on racial microaggressions indicates robust associations with worse adjustment, including greater internalizing problems and stress/negative affect and poorer positive functioning (Lui & Quezada, 2019). For incivility and related low-intensity mistreatment, meta-analyses demonstrate decrements in job satisfaction and commitment, increases in turnover intent, poorer perceived performance/productivity, and elevated depression, exhaustion, and physical health problems; these patterns extend to witnesses of mistreatment as well (Hershcovis & Barling, 2010; Nielsen et al., 2024; Yang et al., 2014). Importantly, declines in job attitudes and efficacy beliefs—such as reduced job satisfaction, organizational commitment, perceived productivity, and self-efficacy—are well-established proximal predictors of disengagement, withdrawal behaviors, and eventual job or career exit (Griffeth et al., 2000; Tett & Meyer, 1993).

### 1.2. Summary and Research Questions

As this review demonstrates, sexual harassment, racial mistreatment, and incivility remain pervasive in biomedical and STEM training environments and disproportionately affect individuals with marginalized identities, including women and racial/ethnic minorities. Such experiences not only undermine psychological and physical health but also threaten trainees’ academic engagement, satisfaction, and long-term participation in the scientific workforce. The present research aims to measure the impact of mistreatment experienced during a very short time frame—one of two days within a 10-day period—a to provide a more precise lens on how often these behaviors occur, how intense they are, and how they shape trainees’ emotional and attitudinal functioning in the short term. This research may point to interventions beyond general policy and training programs that may address these immediate impacts to preclude further harm to targets’ career ambitions.

This inquiry is especially critical because capturing mistreatment as it unfolds over a narrow time window offers clearer insight into the burden trainees carry, illuminates how even isolated or subtle events may contribute to derailment-relevant attitudes, and provides an empirical foundation for efforts to create safer and more equitable training environments. Accordingly, our first study assessed trainees’ single-day experiences and observations of sexual harassment, racial mistreatment, and incivility; examined how these forms of mistreatment clustered together; and evaluated their associations with affective states and program attitudes ten days later. We also explored whether mistreatment differed by the trainee’s gender or racial/URM status, whether its impact varied by experiencing vs. observing mistreatment, and how the gender and leadership status of perpetrators shaped the severity and consequences of these events. In the second study, we sought to understand trainees’ reactions to peers’ accounts of mistreatment and what they believe mentors and institutions should do when such incidents occur.

Guided by these aims, our research addressed the following questions:

1. What is the prevalence and intensity of single-day experiences of sexual harassment, racial mistreatment, and incivility among graduate students and post-doctoral fellows in biomedical health training programs, and do these exposures differ by trainees’ gender and racial/URM status?
2. To what extent do these forms of mistreatment co-occur, and how strongly are they correlated with attitudinal outcomes within a 10-day period (controlling for state positive and negative affect at Time 1)?
3. Do affective and attitudinal outcomes differ depending on whether trainees experienced mistreatment directly, observed it occurring to others, or were exposed in both ways?
4. How does the intensity and consequences of mistreatment vary according to the gender and leadership status of the perpetrator?
5. How do trainees react to peers’ narratives of mistreatment, and what actions do they believe mentors and institutional leaders should take when such events occur?

## 2. Study 1

The purpose of Study 1 was to survey graduate students and post-doctoral fellows in biomedical health training programs in the U.S. on their experiences of mistreatment in the past 24 h. We repeated the survey 10 days later to measure prospective correlations between experiences measured on the first day with program attitudes ten days later.

### 2.1. Study 1 Participants and Procedure

We randomly sampled 2000 graduate students and post-doctoral fellows who had an active fellowship or training grant and were listed in the NIH’s Reporter database (reporter.nih.gov) in 2022. Specifically, we proportionally sampled F30, F31, F32, and K99 awards (M.D./Ph.D., graduate, post-doctoral fellows, and training grants, respectively) in Reporter and invited the awardee by email to participate in a two-part online survey for the chance to win one of ten $100 Amazon gift cards. Each survey was completed on the Qualtrics survey platform (Qualtrics, Provo, UT, USA). We received 404 usable surveys for the first wave (response rate = 20%) and 299 who completed both waves (follow up response rate = 74%). Table 1 provides demographic information about these samples. The composition of the sample at Time 1 and at Time 2 was not significantly different on any of the demographic variables listed in this table (all *p*’s are nonsignificant for *χ*^2^ goodness of fit tests).

Ethical approval for this study was granted by the host university’s institutional review board as exempt protocol #15142. All participants were provided with a study information sheet (similar to an informed consent statement) explaining the procedures, risks and benefits of the study, confidentiality protections, and compensation. Participants were assured that their responses would be confidential and that reports of the findings would not be traceable to them.

### 2.2. Study 1 Measures

In addition to measuring participants’ own and their mentors’ demographic characteristics, we measured participants’ experiences of sexual harassment, racial harassment, racial microaggression, incivility, and allyship using modified versions of existing scales (except as noted below). One purpose of the study was to assess the psychometric qualities of shortened versions of the scales for a daily diary study of a related population (see Kiebler et al., 2026). Because short scales are preferred for such studies to reduce survey fatigue (Gabriel et al., 2019), we developed and assessed shortened measures and assessed their reliability. Although we assessed internal consistency reliability estimates, as noted below, we regarded our experiential measures as formative constructs, meaning that the responses to the items in the scale form the respondents’ standing on the construct (e.g., higher scores equal greater intensity of the experiences). Internal consistency estimates of formative constructs may not be high if the experiences are distinct. By comparison, measures of reflective constructs refer to those where respondents’ standing on the construct causes their responses to the items in the measure (Edwards & Bagozzi, 2000).

Sexual harassment (SH) was measured with a 4-item adaptation of the Sexual Experiences Questionnaire (SEQ; Fitzgerald, 1990; Stark et al., 2002), with items representing each of the four types of sexual harassment experiences captured by the SEQ modified for research lab contexts. Furthermore, we modified the SEQ (and other mistreatment variables described below) by measuring whether they experienced or observed others experiencing the behavior. Our 4-item scale measured (a) sexist hostility (someone in my lab engaged in sexist behavior toward me or others), (b) sexual hostility (someone in my lab engaged in sexually crude behavior toward me or others), (c) unwanted sexual attention (someone in my lab gave me or others unwanted sexual attention), and (d) sexual coercion (someone in my lab implied that I or others would be treated differently if we cooperated sexually with them). Each item was preceded with the stem “please indicate the extent to which you experienced or observed the following events in the past 24 h,” with responses recorded on a scale from 0 (definitely not), 1 (possibly, but not sure), and 2 (yes, definitely). Past research on variants of the SEQ have used three- and five-point scales (e.g., Fitzgerald et al., 1988; Kayaalp et al., 2025; Shannon et al., 2007; Stark et al., 2002). We chose a three-point scale because it seemed unlikely that further gradations of frequencies of mistreatment experiences, such as a few times, often, and very often (Stark et al., 2002), would be relevant for 24 h assessments. Items were summed to create the scale sexual harassment. Time 1, *α* = 0.61; Time 2, *α* = 0.65. Although these reliability estimates were below the conventional cutoff of 0.70, we retained the scales because they form a measure of the frequency of those experiences, not a reflection of an underlying construct.

Racial mistreatment (RM) was measured with two items from a version of the SEQ designed for Latinas (Cortina, 2001), with the same stem as above: In the past 24 h: (a) someone in my lab engaged in racist behavior toward me or others; and (b) someone in my lab engaged in racially crude behavior toward me or others. To these items, we added 5 items representing racial microaggressions that have been discussed in the literature (Nadal, 2011; Sue et al., 2007; Torres-Harding et al., 2012). Using the same stem as above, the items were (a) made assumptions that I or other minorities were inferior; (b) treated me or other minorities as a second-class citizen; (c) invalidated me or other minorities’ experiences as a person of color; (d) was subtly aggressive toward me or other minorities; and (e) ignored me or other minorities or made us feel invisible. Responses to these items were recorded with the same scale anchors as described above. Items were summed to create the scale racial mistreatment. Time 1, *α* = 0.89; Time 2, *α* = 0.93.

Incivility (Inc) was measured with a three-item scale based on Cortina et al.’s measure (Cortina et al., 2001): In the past 24 h: (a) someone in my lab put me down or was condescending to me or others; (b) someone in my lab paid little attention to me or others’ opinions; and (c) someone in my lab addressed me or others in unprofessional terms either publicly or in private. These items were preceded with the same stem described above and same scale anchors. The incivility scale was computed by summing items of this scale. Time 1, *α* = 0.84; Time 2, *α* = 0.85.

A scale totaling each of these events, labeled all negative experiential events (All), was also computed for Time 1 and Time 2. *α* = 0.86 and 0.91, respectively. Additionally, dichotomous prevalence measures for each scale for each time period were created such that participants received a score of 1 if they reported “Probably, not sure” or “Yes, Definitely” to at least one item on the respective scale, and 0 if they did not.

Participants who indicated at least one negative event of sexual harassment, racial harassment, racial microaggression, and/or incivility were asked follow-up questions including: (a) was the experience directed at them or others; (b) who perpetrated the experience (primary research mentor, another lab leader, and other lab member); and (c) the gender of the perpetrator(s). All participants were given an opportunity to tell us more about their experiences.

Mood was measured at Time 1 with state-based measures of positive affect (PA) and negative affect (NA) using the Positive and Negative Affect Schedule (Watson et al., 1988). Participants rated on a scale of 1 (very slightly or not at all) to 5 (very much) the extent to which they had these feelings in the past 24 h. Sample items for PA were enthusiastic and interested (10 items, *α* = 0.92). Sample items for NA were scared and distressed (10 items, *α* = 0.88). Mood was assessed to validate that the negative experiential scales and program attitudes would be significantly correlated with PA and NA in expected directions. These variables were also used as control variables in a regression analysis predicting Time 2 program attitudes with Time 1 negative experiential events.

To capture daily attitudes that are theoretically and empirically linked to persistence versus derailment in graduate and post-doctoral training, we assessed four well-established indicators of retention-relevant program attitudes. Organizational commitment, perceived productivity, self-efficacy, and satisfaction are among the most robust attitudinal and motivational predictors of continued engagement and reduced turnover intentions in work and training environments (Allen & Meyer, 1996; Griffeth et al., 2000; Meyer et al., 2002; Pohlan & Steffes, 2025; Saks, 2006). Each of these constructs has been shown to contribute meaningfully to individuals’ decisions to remain versus withdraw, as higher commitment, satisfaction, confidence in one’s abilities, and perceptions of performance effectiveness are consistently associated with stronger engagement and lower turnover across meta-analytic studies of employee attitudes and retention (Christian et al., 2011; Halbesleben, 2010). Accordingly, program attitudes consisted of four items measuring attitudes toward participants’ commitment and performance in their program within the last 24 h: (a) my commitment to remaining in this program was…, (b) my productivity was…, (c) my confidence in my abilities was…, and (d) my satisfaction in my graduate/post-doc program was…. Responses to these items were made on 5-point scales ranging from 1 (much lower than normal) to 5 (much higher than normal). The scale program attitudes (ProgAtt) was computed by averaging responses to these items. Time 1, *α* = 0.74; and Time 2, *α* = 0.72.

In addition to measures about participants, we also measured the lab gender and lab race context for each participant. Lab gender context assessed the extent to which other members of their lab were mostly the same, or other gender, or balanced, using a scale ranging from 1 (one or very few people of the same gender as me), 2 (slightly fewer people of the same gender as me), 3 (about the same number of people with the same gender as me), 4 (slightly more people of the same gender as me), to 5 (all or almost all of the same gender as me). Past research has shown that the gender context of the work environment affects sexual harassment experiences (Fitzgerald et al., 1997). Similarly, we measured Lab race context with a similar scale. For both measures, higher scores indicate working in a lab environment where other members are similar in gender and race, respectively. Finally, lab size reflected participants’ self-report of the number of trainees in their lab or research program. These variables were measured only at Time 1.

### 2.3. Study 1 Results

#### 2.3.1. Prevalence and Intensity of Negative Experiential Events (Research Question 1)

Table 2 shows the prevalence of experiencing (or witnessing) each form of negative experiential event by gender (other gender not included) and status as an underrepresented minority (Nguyen et al., 2023; URM, where White and Asian, non-Hispanic trainees were classified as non-URMs and all other race/ethnicities were classified as URMs). Across these two 24 h assessments, 11.6% (*n* = 47) of the sample experienced or observed sexual harassment; 14.6% (*n* = 59) experienced or observed racial mistreatment; 32.7% (*n* = 132) experienced or observed incivility; and 36.9% (*n* = 149) experienced or observed at least one negative experiential event.

Capturing the intensity of the experiences, Table 3 provides the means and standard deviations of the Time 1 and Time 2 negative experiential events scales, as well as mood (positive and negative affect) and program attitudes by gender and by URM status. Means of the experience variables (SH, RH, Inc, and All) are means of the sums for these variables. The PA, NA, and ProgAtt scores are the average of means from their respective scales that range from 1 to 5. T-tests comparing women and men (other gender was excluded) and comparing non-URM to URM participants are reported in Table 3. Contrary to past research, there were no gender differences nor differences by URM status in the average intensity of negative experiential events. Positive affect was higher among URM participants than non-URM participants at both time periods; program attitudes were higher among URM participants than non-URM participants at Time 2.

#### 2.3.2. Intercorrelations and Prospective Correlations Among Experiences, Mood, Program Attitudes, and Lab Characteristics (Research Question 2)

To investigate research questions regarding the co-occurrence of negative experiential events and the impact of those events on career-derailing program attitudes, we computed correlations among the negative experiential events measures and the program attitudes scales; to investigate the validation of those scales with positive and negative affect, we correlated those variables with lab characteristics, including lab size, lab gender, and race gender. Furthermore, we examined the prospective correlations between the negative experiential event scales measured at Time 1 with those events at Time 2 (assessing the persistence of the negative experiential events). Table 4 summarizes those analyses.

Among the Time 1 intercorrelations, shown in the bottom diagonal of the correlation matrix in Table 4, and among the Time 2 intercorrelations shown in the upper diagonal, all negative experiential event measures were significantly and positively correlated with each other, indicating that these forms of mistreatment co-occur. Furthermore, the negative experiential events scales were individually and collectively negatively correlated with PA and program attitudes and positively correlated with NA supporting the validity of these scales. All negative experiential events, and program attitudes were prospectively positively correlated with their same scale across the 10-day interval. Lab gender was negatively correlated with the negative experiential events variables, indicating that such experiences were more likely to occur in labs where the focal participant was a gender minority. There were no significant correlations of these variables with lab race or lab size.

We conducted a linear regression to predict Time 2 program attitudes with Time 1 negative experiential events (sexual harassment, racial mistreatment, and incivility), controlling for Time 1 mood (positive and negative affect). There was no evidence of multicollinearity (the variance inflation factors were less than 2.0). The block with mood variables significantly predicted Time 2 program attitudes, *R*^2^ = 0.15, *p* < 0.001. Positive affect positively predicted Time 2 program attitudes, *b* = 0.27 (*SE* = 0.04), *p* < 0.001, and negative affect negatively predicted Time 2 program attitudes, *b* = −0.11 (*SE* = 0.05), *p* = 0.036. The negative experiential events variables contributed incremental variance to the prediction of Time 2 program attitudes, Δ*R*^2^ = 0.03, *p* = 0.013. Time 1 sexual harassment significantly predicted Time 2 program attitudes, *b* = −0.18 (*SE* = 0.08), *p* = 0.019. A second regression analysis was conducted with the mood variables entered in block 1 followed by the composite all negative experiential events variable at Time 1, which did not add significant incremental variance to the prediction of Time 2 program attitudes, Δ*R*^2^ = 0.01, *p* = 0.079, although there was a marginal negative effect of all negative experiential events, *b* = −0.02 (*SE* = 0.01), *p* = 0.079.

#### 2.3.3. Comparison Between Experience Type on Mood and Program Outcomes (Research Question 3)

**Figure 1:**
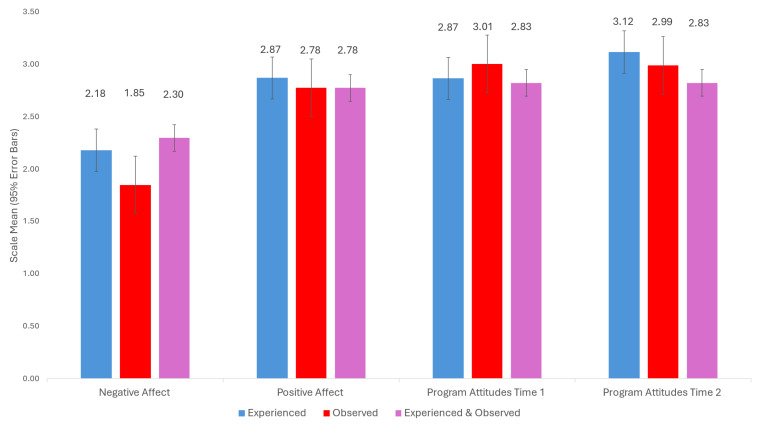
Scale means (95% error bars) of negative affect, positive affect (measured at Time 1), and program attitudes measured at Time 1 and Time 2 by experience type: experienced, observed, or experienced and observed.

Participants who experienced at least one negative experiential event at Time 1 or at Time 2 were grouped into three categories: (a) experienced a negative experiential event (Time 1, *n* = 32; Time 2, *n* = 28); (b) observed a negative experiential event (Time 1, *n* = 44; Time 2, *n* = 26); or experienced and observed a negative experiential event (Time 1, *n* = 53; Time 2, *n* = 15). One-way ANOVAs on program attitudes, positive affect, and negative affect were computed to compare these different experience types. Tukey post hoc tests were conducted to probe significant differences. The means, standard deviations, and F-test results, as well as 95% error bars, are depicted in Figure 1. At Time 1, there were no differences between these groups in program attitudes, *F*(1, 126) = 0.82, *p* = 0.445. or positive affect, *F*(1, 126) = 0.16, *p* = 0.854. Those who both experienced and observed a negative experiential event had significantly higher ratings of negative affect than those who only observed a negative experiential event, *F*(1, 126) = 4.01, *p* = 0.020. The Tukey post hoc test revealed a significant difference between experiencing and both experiencing and observing mistreatment, *p* = 0.017. At Time 2, there were no significant differences between those groups on program attitudes, *F*(1, 91) = 1.38, *p* = 0.257. Overall, these findings indicate that observing a negative experiential event was just as harmful as experiencing it, and if both occurred, the impact on state negative affect was the most consequential.

#### 2.3.4. Characteristics of Perpetrators

Means and standard deviations of the negative experiential events scales by perpetrator characteristics are presented in Table 5. To examine differences in the intensity of negative experiential events and differences in mean scores of positive and negative affect (at Time 1) and of program attitudes by perpetrator characteristics, *t*-tests were conducted comparing respondents who reported a negative experiential event by male or female perpetrators and by perpetrators who were leaders (mentors or other lab leaders) or other lab members. Where Levene’s test for equality of variances was violated, the *t*-test for equal variances not assumed was interpreted. Because the power in these analyses was low in several comparisons (ranging from 0.05 to 0.90 for Time 1 comparisons between male and female perpetrators; between 0.10 and 0.95 for Time 2 comparisons between male and female perpetrators; between 0.05 and 0.69 for Time 1 comparisons between leader vs. non-leader perpetrators; and between 0.05 and 0.58 for Time 2 comparisons between leader vs. non-leader perpetrators), we also examined the effect sizes (Cohen’s *d*), interpreting those that were ≥0.50 (a medium effect size) as meaningful.

At Time 1, sexual harassment perpetrated by men was significantly and meaningfully more intense than sexual harassment perpetrated by women. There were no significant or meaningful differences by gender of the perpetrator on other negative experiential events or on outcome measures. At Time 2, negative experiential events perpetrated by women were more intense than those perpetrated by men on all scales, including the composite all negative experiential events (the effects on sexual harassment and racial mistreatment were marginally significant but demonstrated a meaningful difference per Cohen’s *d* > 0.50). There was a trend for program attitudes at Time 2 to be more positive among participants whose perpetrators were men than among those whose perpetrators were women (see Table 5).

For comparisons between negative experiential events perpetrated by lab leaders and those perpetrated by other lab members at Time 1, there were no significant or meaningful differences in their intensity. However, program attitudes were lower among those whose perpetrators were lab leaders compared to other lab members. At Time 2, sexual harassment, racial mistreatment, and the composite all negative experiential events perpetrated by lab members were more intense than those experiences perpetrated by lab leaders (all *d*’s > 0.50); however, there was no difference in program attitudes scores at Time 2 between leader and non-leader perpetrators (see Table 5).

### 2.4. Study 1 Discussion

Our survey of promising graduate students and post-doctoral fellows who received an NIH fellowship indicated that on one of two 24 h periods, more than a third had a negative experiential event of sexual harassment, racial mistreatment, or incivility, and that those experiences were associated with lowered positive affect, higher negative affect, and negative attitudes toward their program, which could potentially derail their persistence in the biomedical health research enterprise. Moreover, these experiences were intercorrelated, both on a single day, as well as across ten days. We also demonstrated that sexual harassment experienced at Time 1 was negatively associated with program attitudes at Time 2, controlling for positive and negative affect.

Our single-day estimates of harassment and other negative experiential events revealed new findings about gender, race, and power differentials in the experience of and perpetration of such experiences. Unlike past research that has shown that women are more likely than men to experience sexual harassment (Clancy et al., 2014; Wood et al., 2021), and to some extent incivility (Abate & Greenberg, 2023; Mgboji et al., 2021; Moss & Mahmoudi, 2021) in academic environments, and that racial minorities are more likely than others to experience racial harassment and racial microaggressions (Buchanan et al., 2009; Clancy et al., 2017; El Kurd & Hummel, 2023) in such contexts, our findings showed no significant differences in the intensity of these various forms of mistreatment by gender or race of the participant (although there was trend for URM participants to experience more intense racial mistreatment than non-URM participants at Time 1 only, *p* = 0.092). In addition, although past research finds that men are more likely than women to perpetrate sexual harassment in biomedical health academic contexts (Clancy et al., 2014; Phillips et al., 2019; Wood et al., 2021), we found mixed evidence of perpetrator gender differences in participants’ intensity of negative experiential events. At Time 2, participants reported more intense mistreatment experience at the hand of women. Furthermore, at Time 2, negative experiences at the hands of non-leader lab members were more intense (except for incivility) than by lab leaders.

Our contrary findings regarding the gender, race, and status of experiencers and perpetrators may be due to the difference in our methodology. Whereas past research has asked survey participants to recall their negative experiential events over a long period of time, ranging from a year to a lifetime, which may be subject to recall bias (Chawla et al., 2021), our single-day estimates were not likely affected by this bias. It is possible that longer-range recall tasks bias recall in favor of more prototypical and memorable experiences, such that women and people with marginalized identities may remember mistreatment experiences over time, especially those that target their marginalization. It is also possible that perpetration by people with sociocultural power (e.g., men) or structural power (e.g., mentors, leaders) has longer-lasting effects or may persist for a longer time and, thus, be more memorable (Amber et al., 2019). A second explanation is that we measured both experiences and observations of mistreatment. Because anyone could observe a mistreatment, this assessment may have masked identity-based differences in the experience of such mistreatment. Future research should test whether our findings are replicable but also examine how mistreatment impacts unfold over time. Nonetheless, as summarized above, single-day negative experiential events were consequential, leading to an erosion of positive attitudes toward and commitment to staying with their program.

While Study 1 quantified the frequency and short-term correlates of single-day mistreatment, it could not capture how trainees interpret these experiences, how they contextualize them within lab hierarchies, or why even isolated events appear consequential. Accordingly, Study 2 was designed to extend the quantitative findings by exploring participants’ subjective interpretations, perceived mechanisms, and contextual influences surrounding these events.

## 3. Study 2

Past research finds that trainees’ experiences of sexual harassment, racial mistreatment, or incivility are correlated with adverse consequences, such as psychological and physical distress and withdrawal from academic pursuits (Clancy et al., 2014; Cortina et al., 1998; Szymanski & Lewis, 2016). Research also finds that how institutional leaders respond to such claims can either exacerbate or diminish those consequences (Adams-Clark et al., 2019; Hayes & Bigler, 2013; Smith & Freyd, 2017). We sought to understand what trainees wanted their leaders and institutions to do in situations where mistreatment occurs to inform interventions that are centered on their perspectives. To address research question 5, we created immersive narratives of true stories of mistreatment for students to enhance their empathy and perspective taking, and we asked them to describe the impact of those narratives and what they would have wanted their mentors, other leaders, and institutions to have done if those experiences happened to them. We used a grounded theory approach to reveal the themes of their responses to the narratives and our prompts (Strauss & Corbin, 1994).

### 3.1. Overview

Study 2 involved soliciting individuals who had experienced mistreatment in the context of their biomedical health graduate training programs who would be willing to be interviewed and have their interviews transcribed, de-identified, and re-enacted by professional actors to create “empathy stories.” The candidates for these stories came from two sources. One was a previous study conducted by the first author in 2022 who solicited candidates at a Midwestern university by posting flyers around campus. From that effort, four candidates were considered and two were selected for interviews that eventually became two of our empathy stories (pseudonymously Diana and Sarah). The second source came from participants in Study 1 of the current research. We gleaned open-ended comments for participants who agreed to be contacted for potential stories. Again, there were four potential candidates of which two were selected for interviews that became the stories of Sai Li and Brian (pseudonyms).

**Figure 2:**
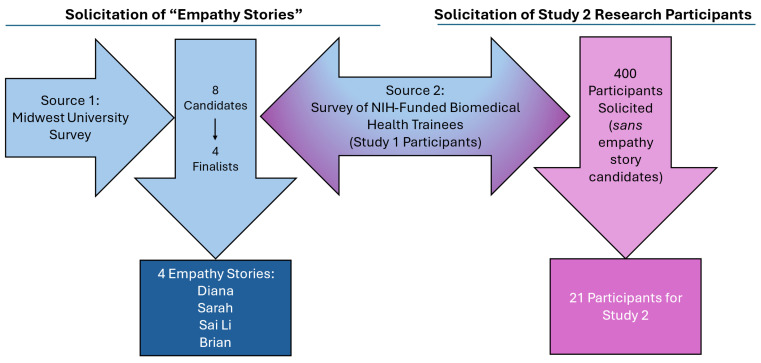
Study 2—Diagram of solicitation process for empathy stories and research participants.

Our research participants for Study 2 were individuals who participated in Study 1 of the current research *sans* the candidates for the empathy stories. Of those who were solicited, 21 agreed to participate in this follow up study. Figure 2 diagrams the solicitation process for both the empathy stories and the participants for Study 2. By recontacting Study 1 participants for this follow up study, we hoped to develop greater insight into how biomedical trainees who had participated in Study 1 feel about the type of mistreatment that had previously been surveyed about. However, by having them respond to other trainees’ stories, we could control the stimulus (empathy story) they were responding to.

### 3.2. Study 2 Participants and Procedure

Graduate students and post-doctoral students who participated in Study 1 who were not candidates for the empathy stories were invited via email to participate in Study 2 for which they would receive a chance to win one of five $50 gift certificates. Twenty-one participants from Study 1 completed Study 2 (52% women, 19% men, 29% did not disclose; 62% White, 9% non-White or multiracial, 29% did not disclose).

Participants were provided with a link to one of four videotaped interviews of similarly situated graduate students who experienced harassment and other forms of mistreatment and a transcript of another one of those four stories (the video and transcript were of different stories), and they responded to open-ended questions after each story. Ethical approval for this study was granted by the host university’s institutional review board as exempt protocol #15142. All participants were provided with a study information sheet explaining the procedures, risks, and benefits of the study, confidentiality protections, and compensation. Participants were assured that their responses would be confidential and that reports of the findings would not be traceable to them.

### 3.3. Study 2 Materials

We elicited narratives of students’ sexual harassment and other mistreatment experiences in previous unpublished research and in Study 1 by soliciting students with those experiences to tell us their stories. In the prior research, we recruited participants at a Midwestern urban university by posting flyers around campus asking if they had experienced sexual harassment and whether they would consent to a confidential interview. A QR code led them to a survey where they briefly described their experience and provided contact information. From Study 1 of the current research, we asked respondents who had experienced at least one form of mistreatment to briefly describe their experience. From those responses from both the previous and current research, we reached out to those who told us about mistreatment experiences that took place in an academic setting and had sufficient detail to discern that they had experienced sexual or racial harassment, microaggression, or incivility. Four respondents from the previous research and four from the current research were invited to be interviewed by the senior author who asked them to describe their experiences in more detail and the impact their experience had on them. From those initial interviews, four (two from each source) were chosen to participate in a longer interview conducted by a graduate student in training to become a clinical psychologist. Those interviews were transcribed, edited, and re-enacted by a professional actor and filmed by a professional videographer. Care was taken to retain the original wording and communication style of the interviewee. These interviews resulted in four “empathy videos” and transcripts to be used for research and training purposes.

Ethical approval for the solicitation of individuals for empathy stories was granted by the host university’s institutional review board as exempt protocols #12914 and #15142. All participants were provided with a study information sheet, and they were assured that their responses would be confidential and that reports of the findings would not be traceable to them. In addition, they were provided with a list of resources such as the National Domestic Violence Hotline and the National Sexual Assault Hotline. The four individuals who completed the interviews that became our empathy stories were compensated with a $200 gift card.

One story, “Diana,” portrayed the interview of a White woman harassed by an external researcher who was allowed to use adjacent lab space. When she complained about the treatment to her mentor and other faculty, nothing was done. Only when a male faculty member complained of bullying by the same person was the perpetrator removed. Diana’s advisor continued to downplay her experience and lost interest in mentoring her. She eventually left the program without completing her Ph.D. The story of “Sarah,” a biochemistry graduate student portrayed by a Black woman, described her experiences of harassment by another person who cornered her in her research lab while her male lab mate looked on. The harasser revisited the lab several times asking Sarah for a date, which she repeatedly turned down. She expressed her fear of being in the lab alone. “Sai Li’s” story, portrayed by an Asian woman, described bullying, incivility, and harassment of herself and another female graduate student by a post-doctoral fellow, for which the faculty mentor, “Mike,” did nothing and downplayed its seriousness. Finally, the video of “Brian,” a science graduate student, portrays relentless unwanted sexual attention and sexual assault by a fellow male graduate student who the mentor described as a “good researcher” and allowed him to expedite his Ph.D. Each video was approximately 10 min in length. In addition to video formats of these stories, written transcripts were also created. Videos of Diana and Sarah and transcripts of Sai Li’s and Brian’s stories were used in the present research. Links to each of these videos and transcripts can be found in the online Supplementary Materials.

For the qualitative study, an open-ended survey asked respondents to describe how they felt and what they learned from story; how they would have responded if the events in the story happened to them; what steps they would have wanted their mentor to take to prevent this from happening, to address if it had happened, to provide support for them, and to prevent future retaliation.

### 3.4. Study 2 Results

A grounded-theory approach to analyzing the open-ended comments was conducted. Treating participants’ responses to each story across all prompts for the story as a single unit of analysis, thematic coding was derived by first developing basic codes and then axial (cross-cutting) codes. Two coders compared the axial codes and agreed to a common set of codes with definitions and examples of phrases that exemplified the code. They independently coded 25% of the responses to check for reliability; Mezzich’s *Κ* (Mezzich et al., 1981) = 0.83. After discussing differences in opinions and coming to mutual agreement, one coder completed the coding of the remaining data.

Themes from this analysis were organized into four categories: reactions to the story, target responses, the psychosocial support respondents wanted, and the instrumental support they wanted. The responses to each of these categories are described below.

#### 3.4.1. Reactions to the Story

Respondents expressed strong emotions and thoughts about the stories. The first type was anger, disgust, and betrayal, expressed in over 25% of the coded responses. An exemplary comment was: “I’m angered and empathetic towards her.”

The second most common type of response found in 25% of the comments were cognitive reactions such as remarking about how familiar the stories were to them or reflections on how insidious harassment can be, e.g., “Very upset because it’s only one of many stories like this that I have heard and Diana did not deserve to get failed by so many people.”

Empathic emotions expressing an emotional connection with the victim were found in approximately 13% of responses, e.g., “Felt understanding and empathy for her situation.”

Finally, depressive emotions expressing sadness and sympathy were found in 11% of responses, e.g., “Saddened, heard too many stories from women in STEM that are similar.”

#### 3.4.2. What Participants Would Want Their Mentor to Do

In response primarily to the prompt about how they would have acted if it happened to them, four themes were found, which mapped on to Knapp and colleagues’ conceptual model of responding to sexual harassment (Knapp et al., 1997), which categorizes responses along dimensions of the focus of response (self vs. initiator-focus) and mode of response (self vs. supported response). Social coping, a self-focused, supportive response, was the most common (approximately 21% of all comments), which reflected intentions to talk to someone about the problem, such as: “I would have told my PI and hoped she would help.”

Avoidance and denial, a self-focused, self-response, was expressed by 15% of responses, such as: “I realistically would avoid [the perpetrator], especially being anywhere alone with him.”

Responses suggesting confrontation, an initiator-focused, self-response (8%), and Advocacy Seeking (8%)—an initiator-focused, supportive response—were exemplified with comments such as: “I don’t generally have an issue speaking out against grossly disturbing behavior no matter who it is;” and “I hope that I would bring it up to HR.”

In addition, we found themes that corresponded with traditional psychosocial and instrumental mentoring strategies (Kram, 1985), described below.

#### 3.4.3. Psychosocial Support

Respondents clearly expressed a need for psychosocial support from their mentor when asked what they would have liked their mentor to do if the events in the story happened to them. Immediate psychosocial support, reflected in 33% of responses, was expressed in comments such as: “I would expect my mentor to listen to my complaint first, and to at the very least confirm my feelings about the situation verbally.”

Proactive long-term psychosocial support (17%) expressed sentiments that the mentor would have a consistent approach to curtailing harassment and mistreatment matters, e.g., “I would expect my mentor to have an idea of what was going on in the lab and who was present and any problems that particular people seem to cause.”

Finally, comments reflecting reactive long-term psychosocial support (13%) centered on desire for mentors to provide long-term emotional support, e.g., “Check-ins about how I was doing; offering to meet and talk.”

#### 3.4.4. Instrumental Support

Similar to the themes for psychosocial support, respondents expressed problem-solving or “instrumental” support immediately, as well as both proactively and reactively in the long term. Immediate instrumental support was the most common theme (32%), e.g., “Remove the individual in question immediately to prevent violence or further harassment during any investigations.”

Proactive long term instrumental support (15%) was expressed in comments such as: “Include something in lab-onboarding about coming to them about harassment; sharing contacts for harassment in the lab/department.”

Comments reflecting reactive long term instrumental support (11%) anticipated how the mentor could manage the possibility of future mistreatment incidents, e.g., “In general, rearrange lab spaces so that 3 individuals are usually present.”

### 3.5. Study 2 Discussion

Study 2 was designed to extend the quantitative findings of Study 1 by illuminating how trainees interpret and make meaning of experiences of sexual harassment, racial mistreatment, and incivility. Whereas Study 1 established that discrete events are associated with short-term shifts in affect and retention-relevant program attitudes, Study 2 clarifies why these events may exert influence even when they appear minor, ambiguous, or isolated. The qualitative findings suggest that mistreatment functions not merely as an interpersonal slight but as a signal about belonging, power, and psychological safety within the lab environment (Cortina et al., 2013).

Participants reacted to the empathy stories as consequential because they shaped expectations about future treatment, altered perceptions of mentor support, and prompted vigilance regarding subsequent interactions. Even when events were subtle or ambiguous, trainees reported engaging in rumination, self-questioning, and anticipatory coping strategies (Cortina & Wasti, 2005). Assertive responses, such as confronting the perpetrator or reporting the incident were relatively rare. Instead, respondents clearly wanted immediate psychosocial and instrumental support from their mentors to step in and resolve the problem and to provide emotional support to trainees who experience mistreatment in their graduate and post-doctoral training. These interpretive processes help explain why, in Study 1, negative affect was elevated following exposure to mistreatment. Importantly, the reactions to empathy stories suggest that the impact of mistreatment is not limited to direct targets; observing events affecting others contributes to perceptions of lab climate and personal vulnerability. Thus, mistreatment appears to operate both as an individual stressor and as a contextual cue that shapes collective sensemaking and underscores the value of addressing both direct and indirect (ambient) experiences of mistreatment.

## 4. General Discussion

Our research on the pervasiveness of harassment, racial mistreatment, and incivility that graduate students and post-doctoral fellows experience on any single day was alarmingly high, agnostic to the gender or race of the student, intercorrelated, and likely to lead to derailing attitudes about their biomedical health training program. Furthermore, when presented with stories of mistreatment that similarly situated peers were experiencing, these trainees were angered and disgusted, and they wanted their mentors to step in.

### 4.1. Research Implications

Prior research has documented that harassment, racial mistreatment, and incivility are prevalent in academic settings and are associated with disengagement, burnout, and attrition risk, but it often relies on long recall windows (months/years) that can mask how frequently these events occur in “ordinary” days and how quickly they shift trainees’ affect and training attitudes (Hu et al., 2019; Schilpzand et al., 2016). By using short time windows (daily experiences and near-term follow-up) and pairing prevalence estimates with affect and program attitudes, the current work sharpens what everyday mistreatment means in practice: it becomes something trainees experience and respond to continuously, not just something captured annually.

This research provides unique insights on the extent of mistreatment occurring in research training programs by estimating the prevalence of those experiences on any single day. Prior research has estimated those experiences over a period of several months to an indefinite period. Our research pinpoints how frequently mistreatment occurs in a very short period of time—one of two days within a 10-day window. Consistent with their conceptualizations as brief, commonplace, low intensity, and ambiguous, racial microaggressions and incivility were the most frequently experienced mistreatments. Yet when asked to tell us more about their experience in their lab or training environment, those describing mistreatment conveyed stories of sexual harassment, from which we developed the empathy videos and transcripts for Study 2. Our findings underscore the importance of examining the antecedents, mediators, and consequences of multiple forms of mistreatment, including harassment, microaggressions, and incivility, and perhaps also abusive supervision, bullying, ostracism and so forth to not only document their prevalence and severity but to also point to possible remedies.

### 4.2. Practical Implications

The findings from these studies have several implications for interventions aimed at reducing negative experiential events in academic settings. First, comprehensive and ongoing training programs are essential to raise awareness about the different forms of mistreatment and their impact. Training should focus not only on potential targets of mistreatment, but also bystanders, and leaders. Barta et al. (2026) in this Special Issue describe an empathy training program that produces positive intentions for proactive bystander intervention. Such training could be expanded to include scripts for how to intervene effectively, articulate ways that concerns can be escalated and provide advice on psychologically safe ways to respond (Zara et al., 2024).

Second, institutions should hold faculty mentors accountable for not only promptly correcting incidents of mistreatment occurring in their programs but more importantly creating conditions that prevent it from happening. Specifically, faculty mentors need tools for fostering inclusive and supportive environments, particularly in lab settings, that can mitigate the prevalence of negative behaviors (see Stockdale et al., 2021). Regular assessments and feedback mechanisms should be implemented to monitor the effectiveness of these interventions and make necessary adjustments. Mentors can also be supported by building the capacities for rapid responses to mistreatment disclosure. Trainees’ reactions to the peer narratives (Study 2) indicate strong expectations that mentors will intervene, which suggests that structured mentor training in culturally responsive ways would be important (Byars-Winston et al., 2018).

Third, psychosocial safety protocols could be added to labs’ other safety protocols to support and sustain a culture of caring (Amoadu et al., 2023; Dunn et al., 2024; Stockdale et al., 2021). These protocols could include “micro-climate checks,” monthly anonymous pulse surveys that track both mistreatment experiences but also changes in affect and attitudes. This operationalizes what incivility research identifies as cumulative harm of repeated low-intensity behaviors (Schilpzand et al., 2016). Findings from these assessments could be flagged for intervention, such as those listed above (empathy training, bystander scripts, psychologically safe responding, and other forms of rapid response).

### 4.3. Limitations

Our research is not without limitations. First, although the sampling frame for Study 1 was based on a random sampling of NIH fellowships and training grants, the response rate was low (albeit in the range of modern survey research). We did not advertise in our recruitment that the study was about harassment and other forms of mistreatment, yet it is possible that students with those experiences were more likely to respond to our survey, which was advertised as a study on mentor–mentee relationships. Second, we used relatively brief measures of mistreatment experiences using items from validated survey instruments that most prototypically reflected the experience type. Yet less prototypical experiences may have been underassessed, suggesting that our prevalence estimates may be lower than if full-item versions of the measured had been used. Similarly, because we asked participants to report on their experiences in the past 24 h. Surveys that were opened on a Sunday or Monday may have underestimated the 24 h prevalence on the assumption that trainees were less likely to be working with others in the research training environments on weekends. Third, the stories that we developed from prior research participants’ reports of mistreatment all described sexual harassment, although there were hints of other forms of mistreatment in some of these stories. Therefore, we cannot be certain that the reactions that participants in Study 2 had to these stories would generalize to racial mistreatment and incivility stories. Future research should collect such stories using the real stories and voices of those who experience them and gather trainees’ reactions to those stories.

### 4.4. Conclusions

This research underscores the urgent need for academic institutions to address the prevalence and impact of harassment, racial mistreatment, and incivility among students in research training environments. By understanding the co-occurrence and persistence of these negative experiential events and their detrimental effects on trainees’ well-being and program attitudes, institutions can implement targeted interventions to create safer and more supportive research training environments. Future research should continue to explore the intersectionality of race and gender in these experiences and develop effective strategies to combat these pervasive issues.

## Figures and Tables

**Table 1:** Study 1 sample characteristics.

Characteristic	Time 1		Time 2	
	N	%	N	%
Gender				
Man	137	34.3%	102	34.1%
Woman	262	64.9%	193	64.5%
Binary/Gender Fluid/Non-Conforming	5	1.2%	4	1.3%
Race/Ethnicity				
Hispanic/Latine (any race)	34	8.4%	24	8.0%
Asian/Asian American, non-Hispanic	54	13.4%	35	11.7%
Black/African American, non-Hispanic	20	5.0%	10	3.3%
White, non-Hispanic	259	64.1%	203	67/9%
Other, non-Hispanic	8	2.0%	8	2.7%
Two or more, non-Hispanic	28	6.9%	19	6.4%
Underrepresented Minority (URM) ^a^	90	22.3%	61	20.4%
U.S. Citizenship				
No	31	7.7%	21	7.0%
Yes	373	92.3%	278	93.0%
NIH Award Type				
F30 (MD/PhD fellowship)	50	12.4%	35	11.7%
F31 (Graduate fellowship)	202	50.0%	151	50.5%
F32 (Post-doctoral fellowship)	85	21.0%	69	23.1%
K99 (Post-doctoral training grant)	65	16.1%	42	14.0%
Unknown	2	0.5%	2	0.7%
Mentor/Principal Investigator Gender ^a^				
Man	248	62.5%	191	63.9%
Woman	149	37.5%	108	36.1%
Mentor/Principal Investigator Race/Ethnicity				
Hispanic, Latino/a	14	3.5%	12	4.0%
Asian/Asian American	44	11.1%	28	9.4%
Black/African American, non-Hispanic	4	1%	2	0.7%
White, non-Hispanic	318	80.7%	244	82.2%
Other, non-Hispanic	10	2.5%	8	2.7%
Not sure	4	1.0%	3	1.0%
Mentor/Principal Investigator Rank				
Assistant Professor	49	12.3%	39	13.0%
Associate Professor	91	22.9%	69	23.1%
Professor	255	64.2%	189	63.2%
Non-Tenure Track Professor	1	0.3%	0	0.0%
Other	2	0.5%	2	0.7%
Lab Size (number of students/fellows in lab)	M = 5.61	SD = 3.05	M = 5.62	SD = 3.02

^a^ URM is defined as identifying as Hispanic, any race, or a race other than White, Asian, or White–Asian.

**Table 2:** Study 1: Prevalence of experiencing or observing sexist hostility, sexual harassment, racial harassment, racial microaggression, incivility or all negative experiential events in a 24 h period by gender and URM status at Time 1, Time 2, or at either time.

Participant Type	Sexual Harassment	Racial Mistreatment	Incivility	All Negative Events
	Time 1			
All (*n* = 403)	8.9%	12.1%	27.2%	32.4%
Men (*n* = 137)	6.6%	10.2%	27.7%	32.1%
Women (*n* = 261)	9.9%	13.0%	27.2%	32.8%
Non-URM (*n* = 331)	8.7%	11.4%	27.4%	32.8%
URM (*n* = 72)	9.7%	15.3%	26.4%	30.6%
	Time 2			
All (*n* = 299)	5.2%	6.2%	14.6%	17.3%
Men (*n* = 102)	4.4%	5.1%	10.9%	13.9%
Women (*n* = 193)	5.7%	6.9%	16.8%	19.5%
Non-URM (*n* = 252)	5.1%	6.3%	16.3%	19.0%
URM (*n* = 47)	5.6%	5.6%	6.9%	9.7%
	Either Time 1 or Time 2			
All (*n* = 299)	11.6%	14.6%	32.7%	36.9%
Men (*n* = 102)	8.0%	10.9%	28.5%	33.6%
Women (*n* = 193)	13.4%	16.4%	35.1%	38.9%
Non-URM (*n* = 252)	11.7%	13.9%	33.4%	37.7%
URM (*n* = 47)	11.1%	18.1%	29.2%	33.3%

Note: URM = underrepresented minority (all Hispanics and any race/ethnicity other than White or Asian).

**Table 3:** Study 1: Means and standard deviations (SD) of study variables measured at Time 1 and Time 2 by gender (men, women) and by URM Status.

	Gender of Participant						URM Status of Participant					
	Men		Women				Non-URM		URM			
	*M*	*SD*	*M*	*SD*	*t* (*p*)	|*d*|	*M*	SD	*M*	*SD*	*t* (*p*)	|*d*|
Time 1 (possible range)	*n* = 137		*n* = 261				*n* = 312		*n* = 91			
SH (0–12)	0.18	0.75	0.16	0.55	0.21 (0.829)	0.02	0.16	0.62	0.19	0.65	−0.36 (0.721)	0.03
RM (0–21)	0.34	1.27	0.48	1.70	−0.82 (0.412)	0.09	0.35	1.29	0.78	2.32	−1.70 * (0.092)	0.29
Inc (0–9)	1.02	2.22	0.85	1.90	0.82 (0.410)	0.09	0.91	2.03	0.93	2.09	−0.09 (0.933)	0.03
All (0–42)	0.78	2.60	1.08	2.86	−0.88 (0.380)	0.02	1.42	3.36	1.90	4.44	−0.94 * (0.343)	0.15
PA (1–5)	3.09	0.77	3.01	0.82	0.89 (0.375)	0.09	2.98	0.80	3.22	0.81	−2.46 (0.014)	0.33
NA (1–5)	1.88	0.80	1.76	0.65	1.60 ^a^ (0.111)	0.18	1.82	0.73	1.78	0.63	0.48 (0.628)	0.11
ProgAtt (1–5)	3.07	0.67	3.07	0.65	0.06 (0.955)	0.01	3.04	0.65	3.16	0.64	−1.48 (0.140)	0.12
Time 2	*n* = 102		*n* = 193				*n* = 252		*n* = 47			
SH (0–12)	0.14	0.63	0.18	0.79	−0.49 (0.627)	0.06	0.14	0.65	0.28	1.08	−0.82 * (0.253)	0.18
RM (0–21)	0.26	1.17	0.38	1.46	−0.68 (0.497)	0.09	0.30	1.20	0.53	2.01	−0.77 * (0.442)	0.29
Inc (0–9)	0.39	1.20	0.44	1.26	−1.07 (0.286)	0.13	0.63	1.26	0.30	1.06	1.32 * (0.191)	0.19
All (0–42)	0.78	2.60	1.08	2.86	−0.88 (0.380)	0.11	0.95	2.52	1.06	3.72	−0.26 (0.793)	0.05
PA (1–5)	3.00	0.74	2.93	0.83	0.73 (0.233)	0.09	2.91	0.79	3.23	0.85	−2.54 (0.012)	0.40
NA (1–5)	1.71	0.69	1.71	0.69	0.04 (0.965)	0.01	1.72	0.67	1.65	0.59	0.69 (0.492)	0.11
ProgAtt (1–5)	3.19	0.66	3.05	0.62	1.76 (0.080)	0.22	3.05	0.62	3.33	0.75	−2.72 (0.007)	0.43

Notes: URM = underrepresented minority, SH = sexual harassment, RM = racial mistreatment, Inc = incivility, All = all negative experiential events, PA = positive affect; NA = negative affect, ProgAtt = program attitudes, |*d*| = absolute value of Cohen’s d (effect size). ^a^ Levine’s Test for Equality of Variances was significant (*p* < 0.05); therefore, the *t*-test and *p*-value reported is for equal variances not assumed. * *p* < 0.05 or lower.

**Table 4:** Study 1: Intercorrelations and prospective correlations among negative experiential events, PA, NA, program attitudes, and lab characteristics at Time 1 and Time 1.

		Time 2 Variables									
	Variable	(SH)	RM)	(Inc)	(All)	(ProgAtt)	(PA)	(NA)	(Lab Gend)	(Lab Race)	(Lab Size)
Time 1 Variables	SH	0.29 **	0.64 **	0.52 **	0.79 **	−0.14 *			−0.03	−0.03	−0.03
	RM	0.60 **	0.70 **	0.60 **	0.90 **	−0.15 *			−0.04	−0.07	0.03
	Inc	0.51 **	0.54 **	0.58 **	0.85 **	−0.22 **			−0.08	−0.02	0.04
	All	0.72 **	0.85 **	0.87 **	0.68 **	−0.20 **			−0.06	−0.05	0.04
	ProgAtt	−0.16 **	−0.19 **	−0.27 **	−0.25 **	0.53 **			0.01	−0.13 *	−0.11
	PA	−0.16 **	−0.17 **	−0.25 **	−0.26 **	0.59 **					
	NA	0.13 **	0.22 **	0.36 **	0.33 **	−0.34 **	−0.29 **				
	Lab Gend	−0.11 *	−0.15 **	−0.16 **	−0.17 **	0.13 *	0.10 *	−0.13 *			
	Lab Race	0.07	−0.04	−0.01	−0.02	−0.04	−0.02	−0.06	−0.00		
	Lab Size	0.02	−0.02	0.03	0.01	0.01	−0.02	−0.03	−0.18 **	0.03	

Notes: Time 1 correlations appear below the diagonal shaded green. Time 2 correlations appear above the diagonal shaded gray. The 10-day prospective correlations between each negative experiential event variable and program attitudes appear in the diagonal, italicized and shaded blue. SH = sexual harassment, RM = racial mistreatment, Inc = incivility, All = all negative experiential events, ProgAtt = program attitudes, PA = positive affect; NA = negative affect, Lab Gend = lab gender. * *p* < 0.05; ** *p* < 0.01 or lower.

**Table 5:** Study 1: Means, SDs of negative experiential events, mood variables, and program attitudes by perpetrator characteristics, Time 1, and Time 2.

Variable	Perpetrator Gender						Perpetrator Leadership Status					
	Male Perpetrators		Female Perpetrators				Mentor or Other Lab Leader Perpetrators		Other Lab Member Perpetrators			
	*M*	*SD*	*M*	*SD*	*t* (*p*)	|*d*|	*M*	*SD*	*M*	*SD*	*t* (*p*)	|*d*|
Time 1	*n* = 42–56		*n* = 22–29				*n* = 24–32		*n* = 42–56			
SH	0.84	1.29	0.21	0.56	3.14 * (0.002)	0.58	0.50	0.95	0.66	1.15	−0.67 (0.504)	0.15
RM	2.20	3.27	1.10	2.06	1.88 * (0.063)	0.38	1.84	2.44	1.77	3.09	0.12 (0.905)	0.00
Inc	3.38	3.33	3.69	3.33	−0.45 (0.652)	0.10	4.09	3.41	3.11	2.75	1.48 (0.142)	0.33
All	6.41	6.09	5.00	4.91	1.08 (0.284)	0.25	6.55	5.52	5.54	5.70	0.72 (0.472)	0.16
PA	2.67	0.80	2.81	0.83	−0.72 (0.474)	0.16	2.48	0.84	2.80	0.80	−1.81 (0.074)	0.40
NA	2.22	0.78	2.26	0.89	−0.22 (0.828)	0.05	2.38	0.88	2.13	0.74	1.41 (0.161)	0.31
ProgAtt	2.81	0.83	2.87	0.77	−0.39 (0.699)	0.09	2.51	0.88	2.95	0.62	−2.75 (0.007)	0.61
Time 2	*n* = 15		*n* = 20				*n* = 12		*n* = 29			
SH	0.47	0.64	1.30	1.95	−1.79 * (0.086)	0.54	0.25	0.45	1.10	1.78	−2.40 * (0.022)	0.56
RM	0.73	1.33	2.30	3.28	−1.93 * (0.064)	0.60	0.58	1.24	2.21	2.96	−2.48 * (0.018)	0.63
Inc	1.40	1.35	3.55	2.06	−3.71 * (<0.001)	1.20	2.00	2.04	2.62	2.14	−0.85 (0.398)	0.29
All	2.60	1.72	7.15	5.94	−3.25 * (0.004)	0.98	2.83	3.04	5.96	5.57	−2.28 * (0.029)	0.62
ProgAtt	3.18	0.75	2.73	0.72	1.83 (0.076)	0.63	2.96	0.88	2.95	0.70	0.04 (0.969)	0.01

Notes: SH = sexual harassment, RM = racial mistreatment, Inc = incivility, All = all negative experiential events, ProgAtt = program attitudes, PA = positive affect; NA = negative affect, |*d*| = absolute value of Cohen’s d effect size. * Levine’s test of homogeneity of variance was significant; therefore, the *t* (*p*) for “equal variances not assumed” was reported.

## Data Availability

Data for Study 1 and Study 2 may be found in the online Supplementary Materials.

## Supplementary Materials

The following supporting information can be downloaded at: https://www.mdpi.com/article/10.3390/bs16030380/s1, Supplementary Files: (a) informed consent statements for Study 1 and Study 2; (b) data collection instruments for Study 1 and Study 2: (c) Links to the videos used for Study 2; (d) datasets for Study 1 and Study 2.

## Abbreviations

The following abbreviations are used in this manuscript:

AAU	American Association of Universities
All	All negative experiential events
EEOC	Equal Employment Opportunity Commission
Inc	Incivility
IRB	Institutional Review Board
Lab Gend	Lab Gender
M.S./Ph.D.	Medical Doctorate/Doctor of Philosophy
NA	Negative Affect
NIH	National Institutes of Health
PA	Positive Affect
ProgAtt	Program Attitudes
RM	Racial mistreatment
SEQ	Sexual Experiences Questionnaire
SH	Sexual Harassment
STEM	Science, technology, engineering, mathematics
URM	Underrepresented minority
