# Supplementary material for: Prevalence and Impact of Single-Day Events of Sexual Harassment, Racial Mistreatment, and Incivility on Biomedical Health Trainees: A Mixed-Methods Study
Source: Behav Sci (Basel). 2026 Mar 6;16(3):380. doi: 10.3390/bs16030380 (PMC13024630; doi:10.3390/bs16030380)
Supplement: Supplementary file 1 [file behavsci-16-00380-s001.zip › Supplementary Files/Informed consent Protocol 12914.pdf]

INDIANA UNIVERSITY

APPROVAL LETTER

To: Stockdale, Peggy

Protocol #: 12914

Protocol Title: Stop Harassment! Harassment interviews

Type of Submission: Initial

Level of Review: Exempt

Approval Date: Monday, November 1st 2021

Expiration Date: no date provided

**If Expiration Date = "No date provided," this research does not require annual renewal; thus there is no expiration date.*

The Indiana University HRPP approved the above-referenced submission. Conduct of this study is subject to the IU HRPP Policies, as applicable.

Additional Notes:

This research is exempt under the following category, and the IRB conducted a limited IRB review: Category 2(iii)

Documents approved with this submission:

Attachments

Study Information Sheet	Stop Harassment SIS.docx
Recruitment Materials	Recruitment Flyer_revised.pdf
Recruitment Materials	Part 2 recruitment email.docx
Recruitment Materials	Recruitment message_Part 3.docx
Data Collection Instrument	SH Online Survey.docx
Data Collection Instrument	SH phone screening questionnaire.docx
Data Collection Instrument	SH interview questions-revised.docx
Other	Resources for SH.pdf

You should retain a copy of this letter and all associated approved study documents in your research records.

If you have any questions or require further information, please contact the HRPP via email at irb@iu.edu or via phone at (317) 274-8289.
