# Supplementary material for: Prevalence and Impact of Single-Day Events of Sexual Harassment, Racial Mistreatment, and Incivility on Biomedical Health Trainees: A Mixed-Methods Study
Source: Behav Sci (Basel). 2026 Mar 6;16(3):380. doi: 10.3390/bs16030380 (PMC13024630; doi:10.3390/bs16030380)
Supplement: Supplementary file 1 [file behavsci-16-00380-s001.zip › Supplementary Files/Informed consent Protocol 15142.pdf]

INDIANA UNIVERSITY

APPROVAL LETTER

To: Stockdale, Peggy

Protocol #: 15142

Protocol Title: Biomedical Research mentor/mentee study

Type of Submission: Initial

Level of Review: Exempt

Approval Date: Thursday, April 21st 2022

Expiration Date: no date provided

**If Expiration Date = "No date provided," this research does not require annual renewal; thus there is no expiration date.*

The Indiana University HRPP approved the above-referenced submission. Conduct of this study is subject to the IU HRPP Policies, as applicable.

Additional Notes:

This research is exempt under the following category:
- Category 4(i)
This research is exempt under the following category, and the IRB conducted a limited IRB review:
- Category 2(iii)

Documents approved with this submission:

Attachments

Data Collection Instrument	Mentor enrollment survey.docx
Data Collection Instrument	Mentor Intake survey.docx
Data Collection Instrument	Mentor daily survey.docx
Data Collection Instrument	Mentee intake survey.docx
Data Collection Instrument	Mentee daily survey.docx
Data Collection Instrument	Secondary Mentee survey.docx
Study Information Sheet	Study Information Sheet - Mentors.docx
Study Information Sheet	Study Information Sheet - Mentees.docx
Study Information Sheet	Study Information Sheet - Secondary Mentees.docx
Recruitment Materials	Mentor recruitment messages.docx
Recruitment Materials	Mentee recruitment messages.docx
Recruitment Materials	Secondary Mentee recruitment messages.docx

You should retain a copy of this letter and all associated approved study documents in your research records.

If you have any questions or require further information, please contact the HRPP via email at irb@iu.edu or via phone at (317) 274-8289.
