# Supplementary material for: Prevalence and Impact of Single-Day Events of Sexual Harassment, Racial Mistreatment, and Incivility on Biomedical Health Trainees: A Mixed-Methods Study
Source: Behav Sci (Basel). 2026 Mar 6;16(3):380. doi: 10.3390/bs16030380 (PMC13024630; doi:10.3390/bs16030380)
Supplement: Supplementary file 1 [file behavsci-16-00380-s001.zip › Supplementary Files/Study 1 data Codebook.pdf]

Prevalence and Impact of Single-Day Events of Sexual Harassment, Racial Mistreatment, and Incivility on Biomedical Health Trainees: A Mixed-Methods Study

Margaret S. Stockdale [1*], Ann C. Kimble-Hill [2], Amanda E. Mosier [1,3], Jessica Kiebler [1,4], Breianna Mildor [1,5] and Darius Washington [1]

Study 1 Data: **Codebook**

Activity

		Value	Count	Percent
Standard Attributes	Position	18		
	Label	NIH Fellowship activity type		
	Type	String		
	Format	A2000		
	Measurement	Nominal		
	Role	Input		
Valid Values			3	0.7%
	F30		50	12.4%
	F31		201	49.8%
	F32		85	21.0%
	K99		65	16.1%

Dem_Citizen

		Value	Count	Percent
Standard Attributes	Position	13		

	Label	Are you an American citizen?		
	Type	Numeric		
	Format	F40		
	Measurement	Scale		
	Role	Input		
N	Valid	403		
	Missing	1		
Central Tendency and Dispersion	Mean	1.08		
	Standard Deviation	.267		
	Percentile 25	1.00		
	Percentile 50	1.00		
	Percentile 75	1.00		
Labeled Values	1	Yes	372	92.1%
	2	No (if no, what is your country of citizenship?)	31	7.7%

Dem_Citizen_No_Text

		Value	Count	Percent
Standard Attributes	Position	14		
	Label	Are you an American citizen? - No (if no, what is your country of citizenship?) - Text		

	Type	String		
	Format	A2000		
	Measurement	Nominal		
	Role	Input		
Valid Values			376	93.1%
	Canada		1	0.2%
	china		1	0.2%
	China		2	0.5%
	Colombia		1	0.2%
	Germany		2	0.5%
	Greece		1	0.2%
	India		6	1.5%
	INDIA		1	0.2%
	Iran		1	0.2%
	Italy		3	0.7%
	Korea		1	0.2%
	Netherlands		1	0.2%
	Peru		1	0.2%
	Russia		1	0.2%
	Taiwan		2	0.5%
	U.K.		1	0.2%
	Ukraine		1	0.2%
	Venezuela		1	0.2%

Dem_Degree_Tenure

		Value
Standard Attributes	Position	16
	Label	Indicate the number of years (to the nearest whole number) that you have: - Been pursuing this degree
	Type	Numeric
	Format	F40.2
	Measurement	Scale
	Role	Input
N	Valid	356
	Missing	48
Central Tendency and Dispersion	Mean	4.4129
	Standard Deviation	1.97042
	Percentile 25	4.0000
	Percentile 50	5.0000
	Percentile 75	6.0000

Dem_DegreeSeeking

		Value	Count	Percent
Standard Attributes	Position	15		

	Label	What degree are you currently seeking?		
	Type	Numeric		
	Format	F40		
	Measurement	Scale		
	Role	Input		
N	Valid	366		
	Missing	38		
Central Tendency and Dispersion	Mean	14.95		
	Standard Deviation	5.287		
	Percentile 25	11.00		
	Percentile 50	11.00		
	Percentile 75	23.00		
Labeled Values	1	BA/BS or equivalent	0	0.0%
	2	MA	0	0.0%
	3	MS	1	0.2%
	4	MPH	0	0.0%
	5	MFA	0	0.0%
	6	MBA	0	0.0%
	7	MLS	0	0.0%
	8	MPH	0	0.0%
	9	MSW	0	0.0%
	10	MArch	0	0.0%

	11	Ph.D.	200	49.5%
	12	PsyD	0	0.0%
	13	M.D.	3	0.7%
	14	MD/PH.D	54	13.4%
	15	DSW	0	0.0%
	16	JD	0	0.0%
	17	DDS	2	0.5%
	18	DDM	0	0.0%
	19	DVM	0	0.0%
	20	DPH	0	0.0%
	21	DEng	0	0.0%
	22	Ed.D	0	0.0%
	23	Other	106	26.2%
	24	Combined bachelors and master's degree	0	0.0%

Dem_Gend

		Value	Count	Percent
Standard Attributes	Position	2		
	Label	What is your gender? - Selected Choice		
	Type	Numeric		
	Format	F40		

	Measurement	Scale		
	Role	Input		
N	Valid	403		
	Missing	1		
Central Tendency and Dispersion	Mean	1.67		
	Standard Deviation	.515		
	Percentile 25	1.00		
	Percentile 50	2.00		
	Percentile 75	2.00		
Labeled Values	1	Male	137	33.9%
	2	Female	262	64.9%
	3	Non-Binary/Genderfluid	3	0.7%
	4	Prefer not to say	0	0.0%
	5	Prefer to self-describe	1	0.2%

Dem_Gend_TEXT

		Value	Count	Percent
Standard Attributes	Position	3		
	Label	What is your gender? - Prefer to self-describe - Text		
	Type	String		
	Format	A2000		

	Measurement	Nominal		
	Role	Input		
Valid Values			403	99.8%
	gender non-conforming woman		1	0.2%

Dem_Hispanic

		Value	Count	Percent
Standard Attributes	Position	5		
	Label	Are you of Hispanic, Latine, or Spanish origin?		
	Type	Numeric		
	Format	F40		
	Measurement	Scale		
	Role	Input		
N	Valid	402		
	Missing	2		
Central Tendency and Dispersion	Mean	1.90		
	Standard Deviation	.300		
	Percentile 25	2.00		
	Percentile 50	2.00		
	Percentile 75	2.00		
Labeled Values	1	Yes	40	9.9%
	2	No	362	89.6%

Dem_Mentor_Tenure

		Value
Standard Attributes	Position	17
	Label	Indicate the number of years (to the nearest whole number) that you have: - Been working in the lab (or under the mentorship) of your primary research mentor
	Type	Numeric
	Format	F40.2
	Measurement	Scale
	Role	Input
N	Valid	399
	Missing	5
Central Tendency and Dispersion	Mean	4.0251
	Standard Deviation	1.52348
	Percentile 25	3.0000
	Percentile 50	4.0000
	Percentile 75	5.0000

Dem_Race_AmerInd

		Value	Count	Percent
Standard Attributes	Position	6		
	Label	American Indian, Native American, First Peoples		
	Type	Numeric		
	Format	F40		
	Measurement	Scale		
	Role	Input		
N	Valid	7		
	Missing	397		
Central Tendency and Dispersion	Mean	1.00		
	Standard Deviation	.000		
	Percentile 25	1.00		
	Percentile 50	1.00		
	Percentile 75	1.00		
Labeled Values	1	American Indian, Native American, First Peoples	7	1.7%

Dem_Race_Asian

		Value	Count	Percent
Standard Attributes	Position	7		
	Label	Asian, Asian American		

	Type	Numeric		
	Format	F40		
	Measurement	Scale		
	Role	Input		
N	Valid	65		
	Missing	339		
Central Tendency and Dispersion	Mean	1.00		
	Standard Deviation	.000		
	Percentile 25	1.00		
	Percentile 50	1.00		
	Percentile 75	1.00		
Labeled Values	1	Asian, Asian American	65	16.1%

Dem_Race_Black

		Value	Count	Percent
Standard Attributes	Position	8		
	Label	Black, African American, African Caribbean		
	Type	Numeric		
	Format	F40		
	Measurement	Scale		
	Role	Input		
N	Valid	27		

	Missing	377		
Central Tendency and Dispersion	Mean	1.00		
	Standard Deviation	.000		
	Percentile 25	1.00		
	Percentile 50	1.00		
	Percentile 75	1.00		
Labeled Values	1	Black, African American, African Caribbean	27	6.7%

Dem_Race_Hawi

		Value	Count	Percent
Standard Attributes	Position	9		
	Label	Native Hawaiian, Pacific Islander		
	Type	Numeric		
	Format	F40		
	Measurement	Scale		
	Role	Input		
N	Valid	3		
	Missing	401		
Central Tendency and Dispersion	Mean	1.00		
	Standard Deviation	.000		
	Percentile 25	1.00		
	Percentile 50	1.00		

	Percentile 75	1.00		
Labeled Values	1	Native Hawaiian, Pacific Islander	3	0.7%

Dem_Race_Other

		Value	Count	Percent
Standard Attributes	Position	11		
	Label	Race Other		
	Type	Numeric		
	Format	F40		
	Measurement	Scale		
	Role	Input		
N	Valid	22		
	Missing	382		
Central Tendency and Dispersion	Mean	1.00		
	Standard Deviation	.000		
	Percentile 25	1.00		
	Percentile 50	1.00		
	Percentile 75	1.00		
Labeled Values	1	Other	22	5.4%

Dem_Race_Other_Text

	Value	Count	Percent

Standard Attributes	Position	12		
	Label	Race Other - Text		
	Type	String		
	Format	A2000		
	Measurement	Nominal		
	Role	Input		
Valid Values			393	97.3%
	Armenian		1	0.2%
	Caribbean-American		1	0.2%
	Hmong		1	0.2%
	I do not identify as white		1	0.2%
	Iranian		1	0.2%
	MENA		1	0.2%
	Middle Eastern		4	1.0%
	mix race		1	0.2%

Dem_Race_White

		Value	Count	Percent
Standard Attributes	Position	10		
	Label	White		
	Type	Numeric		
	Format	F40		
	Measurement	Scale		
	Role	Input		

N	Valid	307		
	Missing	97		
Central Tendency and Dispersion	Mean	1.00		
	Standard Deviation	.000		
	Percentile 25	1.00		
	Percentile 50	1.00		
	Percentile 75	1.00		
Labeled Values	1	White	307	76.0%

Dem_Tran

		Value	Count	Percent
Standard Attributes	Position	4		
	Label	Do you identify as transgender?		
	Type	Numeric		
	Format	F40		
	Measurement	Scale		
	Role	Input		
N	Valid	403		
	Missing	1		
Central Tendency and Dispersion	Mean	2.00		
	Standard Deviation	.070		
	Percentile 25	2.00		
	Percentile 50	2.00		

	Percentile 75	2.00		
Labeled Values	1	Yes	2	0.5%
	2	No	401	99.3%

ID

		Value
Standard Attributes	Position	1
	Label	ID
	Type	String
	Format	A2000
	Measurement	Nominal
	Role	Input

Incivility_1_Day1

		Value	Count	Percent
Standard Attributes	Position	53		
	Label	In the past 24 hours, Someone in my lab put me down or was condescending to me or others.		
	Type	Numeric		
	Format	F40		

	Measurement	Scale		
	Role	Input		
N	Valid	403		
	Missing	1		
Central Tendency and Dispersion	Mean	.26		
	Standard Deviation	.607		
	Percentile 25	.00		
	Percentile 50	.00		
	Percentile 75	.00		
Labeled Values	0	Definitely not	331	81.9%
	1	Possibly, but not sure	39	9.7%
	2	Yes, definitely	32	7.9%

Incivility_1_Day10

		Value	Count	Percent
Standard Attributes	Position	87		
	Label	Incivility Item 1: Someone in my lab put me down or was condescending to me or others		
	Type	Numeric		
	Format	F40		
	Measurement	Scale		

	Role	Input		
N	Valid	299		
	Missing	105		
Central Tendency and Dispersion	Mean	.17		
	Standard Deviation	.469		
	Percentile 25	.00		
	Percentile 50	.00		
	Percentile 75	.00		
Labeled Values	0	Definitely not	261	64.6%
	1	Possibly, but not sure	26	6.4%
	2	Yes, definitely	12	3.0%

Incivility_1_target_Day10

		Value	Count	Percent
Standard Attributes	Position	90		
	Label	Target of behavior: Me		
	Type	Numeric		
	Format	F40		
	Measurement	Scale		
	Role	Input		
N	Valid	299		
	Missing	105		

Central Tendency and Dispersion	Mean	.13		
	Standard Deviation	.337		
	Percentile 25	.00		
	Percentile 50	.00		
	Percentile 75	.00		
Labeled Values	1	Me	39	9.7%

Incivility_2_Day1

		Value	Count	Percent
Standard Attributes	Position	54		
	Label	In the past 24 hours, Someone in my lab paid little attention to my or others' opinions.		
	Type	Numeric		
	Format	F40		
	Measurement	Scale		
	Role	Input		
N	Valid	403		
	Missing	1		
Central Tendency and Dispersion	Mean	.32		
	Standard Deviation	.639		
	Percentile 25	.00		
	Percentile 50	.00		

	Percentile 75	.00		
Labeled Values	0	Definitely not	310	76.7%
	1	Possibly, but not sure	57	14.1%
	2	Yes, definitely	35	8.7%

Incivility_2_Day10

		Value	Count	Percent
Standard Attributes	Position	88		
	Label	Incivility Item 2: Someone in my lab paid little attention to my or others opinions		
	Type	Numeric		
	Format	F40		
	Measurement	Scale		
	Role	Input		
N	Valid	299		
	Missing	105		
Central Tendency and Dispersion	Mean	.21		
	Standard Deviation	.524		
	Percentile 25	.00		
	Percentile 50	.00		
	Percentile 75	.00		

Labeled Values	0	Definitely not	252	62.4%
	1	Possibly, but not sure	31	7.7%
	2	Yes, definitely	16	4.0%

Incivility_2_target_Day10

		Value	Count	Percent
Standard Attributes	Position	91		
	Label	Target of behavior: Others		
	Type	Numeric		
	Format	F40		
	Measurement	Scale		
	Role	Input		
N	Valid	299		
	Missing	105		
Central Tendency and Dispersion	Mean	.10		
	Standard Deviation	.296		
	Percentile 25	.00		
	Percentile 50	.00		
	Percentile 75	.00		
Labeled Values	1	Other	29	7.2%

Incivility_3_Day1

		Value	Count	Percent
Standard Attributes	Position	55		
	Label	In the past 24 hours, Someone in my lab addressed me or others in unprofessional terms either publicly or in private.		
	Type	Numeric		
	Format	F40		
	Measurement	Scale		
	Role	Input		
N	Valid	403		
	Missing	1		
Central Tendency and Dispersion	Mean	.13		
	Standard Deviation	.443		
	Percentile 25	.00		
	Percentile 50	.00		
	Percentile 75	.00		
Labeled Values	0	Definitely not	364	90.1%
	1	Possibly, but not sure	25	6.2%
	2	Yes, definitely	13	3.2%

Incivility_3_Day10

		Value	Count	Percent
Standard Attributes	Position	89		
	Label	Incivility Item 3: Someone in my lab addressed me or others in unprofessional terms either publicly or in private		
	Type	Numeric		
	Format	F40		
	Measurement	Scale		
	Role	Input		
N	Valid	299		
	Missing	105		
Central Tendency and Dispersion	Mean	.11		
	Standard Deviation	.402		
	Percentile 25	.00		
	Percentile 50	.00		
	Percentile 75	.00		
Labeled Values	0	Definitely not	274	67.8%
	1	Possibly, but not sure	16	4.0%
	2	Yes, definitely	9	2.2%

LAB_GEND

		Value	Count	Percent
Standard Attributes	Position	64		
	Label	How many graduate students and/or postdoctoral fellows are working with your primary research mentor? - Selected Choicemale/female dominated		
	Type	Numeric		
	Format	F40		
	Measurement	Scale		
	Role	Input		
N	Valid	377		
	Missing	27		
Central Tendency and Dispersion	Mean	3.38		
	Standard Deviation	1.245		
	Percentile 25	3.00		
	Percentile 50	3.00		
	Percentile 75	5.00		
Labeled Values	1	One or very few people of the same gender as me	36	8.9%

	2	Slightly fewer people of the same gender as me	42	10.4%
	3	About the same number of people with the same gender as me and people with a different gender than me	139	34.4%
	4	Slightly more people of the same gender as me	63	15.6%
	5	All or almost all of the same gender as me	97	24.0%

Lab_GenderMentor

		Value	Count	Percent
Standard Attributes	Position	20		
	Label	What is the gender of your primary research mentor?		
	Type	Numeric		
	Format	F40		
	Measurement	Scale		
	Role	Input		
N	Valid	403		

	Missing	1		
Central Tendency and Dispersion	Mean	1.37		
	Standard Deviation	.485		
	Percentile 25	1.00		
	Percentile 50	1.00		
	Percentile 75	2.00		
Labeled Values	1	Male	252	62.4%
	2	Female	151	37.4%
	3	Non-binary/Genderfluid/Other	0	0.0%

Lab_HispanicMentor

		Value	Count	Percent
Standard Attributes	Position	21		
	Label	Is your primary research mentor of Hispanic, Latine, or Spanish origin?		
	Type	Numeric		
	Format	F40		
	Measurement	Scale		
	Role	Input		
N	Valid	403		
	Missing	1		

Central Tendency and Dispersion	Mean	1.97		
	Standard Deviation	.204		
	Percentile 25	2.00		
	Percentile 50	2.00		
	Percentile 75	2.00		
Labeled Values	1	Yes	14	3.5%
	2	No	386	95.5%
	3	Not sure	3	0.7%

Lab_MentorRace_AmerIn

		Value	Count	Percent
Standard Attributes	Position	22		
	Label	Mentor is American Indian, Native American, First Peoples		
	Type	Numeric		
	Format	F40		
	Measurement	Scale		
	Role	Input		
N	Valid	0		
	Missing	404		
Central Tendency and Dispersion	Mean	.		
	Standard Deviation	.		
	Percentile 25	.		

	Percentile 50	.		
	Percentile 75	.		
Labeled Values	1	American Indian, Native American, First Peoples	0	0.0%

Lab_MentorRace_Asian

		Value	Count	Percent
Standard Attributes	Position	23		
	Label	Mentor is Asian, Asian American		
	Type	Numeric		
	Format	F40		
	Measurement	Scale		
	Role	Input		
N	Valid	44		
	Missing	360		
Central Tendency and Dispersion	Mean	1.00		
	Standard Deviation	.000		
	Percentile 25	1.00		
	Percentile 50	1.00		
	Percentile 75	1.00		
Labeled Values	1	Asian, Asian American	44	10.9%

Lab_MentorRace_Black

		Value	Count	Percent
Standard Attributes	Position	24		
	Label	Mentor is Black, African American, African Caribbean		
	Type	Numeric		
	Format	F40		
	Measurement	Scale		
	Role	Input		
N	Valid	4		
	Missing	400		
Central Tendency and Dispersion	Mean	1.00		
	Standard Deviation	.000		
	Percentile 25	1.00		
	Percentile 50	1.00		
	Percentile 75	1.00		
Labeled Values	1	Black, African American, African Caribbean	4	1.0%

Lab_MentorRace_Hawi

		Value	Count	Percent
Standard Attributes	Position	25		

	Label	Mentor is Native Hawaiian, Pacific Islander		
	Type	Numeric		
	Format	F40		
	Measurement	Scale		
	Role	Input		
N	Valid	0		
	Missing	404		
Central Tendency and Dispersion	Mean	.		
	Standard Deviation	.		
	Percentile 25	.		
	Percentile 50	.		
	Percentile 75	.		
Labeled Values	1	Native Hawaiian, Pacific Islander	0	0.0%

Lab_MentorRace_Notsure

		Value	Count	Percent
Standard Attributes	Position	28		
	Label	Mentor is Race Not sure		
	Type	Numeric		
	Format	F40		
	Measurement	Scale		

	Role	Input		
N	Valid	9		
	Missing	395		
Central Tendency and Dispersion	Mean	1.00		
	Standard Deviation	.000		
	Percentile 25	1.00		
	Percentile 50	1.00		
	Percentile 75	1.00		
Labeled Values	1	Not sure	9	2.2%

Lab_MentorRace_Other

		Value	Count	Percent
Standard Attributes	Position	27		
	Label	Mentor is Other race		
	Type	Numeric		
	Format	F40		
	Measurement	Scale		
	Role	Input		
N	Valid	12		
	Missing	392		
Central Tendency and Dispersion	Mean	1.00		
	Standard Deviation	.000		
	Percentile 25	1.00		

	Percentile 50	1.00		
	Percentile 75	1.00		
Labeled Values	1	Other	12	3.0%

Lab_MentorRace_Text

		Value	Count	Percent
Standard Attributes	Position	29		
	Label	Mentor is Race Text		
	Type	String		
	Format	A2000		
	Measurement	Nominal		
	Role	Input		
Valid Values			396	98.0%
	Afghan		1	0.2%
	Arab		1	0.2%
	Arab/Israeli		1	0.2%
	French		1	0.2%
	Indian		1	0.2%
	Indian, as in from india		1	0.2%
	Iranian		1	0.2%
	More than one race, White/Asian		1	0.2%

Lab_MentorRace_White

		Value	Count	Percent
Standard Attributes	Position	26		
	Label	Mentor is White		
	Type	Numeric		
	Format	F40		
	Measurement	Scale		
	Role	Input		
N	Valid	334		
	Missing	70		
Central Tendency and Dispersion	Mean	1.00		
	Standard Deviation	.000		
	Percentile 25	1.00		
	Percentile 50	1.00		
	Percentile 75	1.00		
Labeled Values	1	White	334	82.7%

Lab_MentorRank

		Value	Count	Percent
Standard Attributes	Position	30		
	Label	Mentor's rank is		
	Type	Numeric		
	Format	F40		
	Measurement	Scale		
	Role	Input		

N	Valid	403		
	Missing	1		
Central Tendency and Dispersion	Mean	2.54		
	Standard Deviation	.730		
	Percentile 25	2.00		
	Percentile 50	3.00		
	Percentile 75	3.00		
Labeled Values	1	Assistant Professor	50	12.4%
	2	Associate Professor	92	22.8%
	3	Full Professor	258	63.9%
	4	Non Tenure Track Professor	1	0.2%
	5	Other	2	0.5%

Lab_MentorRank_Text

		Value	Count	Percent
Standard Attributes	Position	31		
	Label	Mentor's rank is Other - Text		
	Type	String		
	Format	A2000		
	Measurement	Nominal		
	Role	Input		
Valid Values			402	99.5%

	Distinguished Professor		1	0.2%
	Scientific Director		1	0.2%

LAB_RACE

		Value	Count	Percent
Standard Attributes	Position	19		
	Label	In this lab, or under your primary research mentor's mentorship, there are:		
	Type	Numeric		
	Format	F40		
	Measurement	Scale		
	Role	Input		
N	Valid	377		
	Missing	27		
Central Tendency and Dispersion	Mean	2.78		
	Standard Deviation	1.392		
	Percentile 25	1.00		
	Percentile 50	3.00		
	Percentile 75	4.00		
Labeled Values	1	One or very few people of the same race/ethnicity as me	107	26.5%

	2	Slightly fewer people of the same race/ethnicity as me	44	10.9%
	3	About the same number of people with the same race/ethnicity as me and people with a different race/ethnicity than me	101	25.0%
	4	Slightly more people of the same race/ethnicity as me	75	18.6%
	5	All or almost all of the same race/ethnicity as me	50	12.4%

LAB_SIZE

		Value	Count	Percent
Standard Attributes	Position	63		
	Label	How many graduate students and/or postdoctoral fellows are working with your primary research mentor? - Selected Choice		

	Type	Numeric		
	Format	F40		
	Measurement	Scale		
	Role	Input		
N	Valid	403		
	Missing	1		
Central Tendency and Dispersion	Mean	6.25		
	Standard Deviation	5.120		
	Percentile 25	3.00		
	Percentile 50	5.00		
	Percentile 75	8.00		
Labeled Values	1	Only me	26	6.4%
	2	2	35	8.7%
	3	3	55	13.6%
	4	4	63	15.6%
	5	5	46	11.4%
	6	6	35	8.7%
	7	7	35	8.7%
	8	8	24	5.9%
	9	9	16	4.0%
	10	10	20	5.0%
	11	More than 10 (how many?)	14	3.5%

Lab_TimeLabLead_Day1

		Value	Count	Percent
Standard Attributes	Position	40		
	Label	How much time did you spend with a lab leader other than your primary research mentor, such as a lab supervisor or lab manager in the past 24 hours?		
	Type	Numeric		
	Format	F40		
	Measurement	Scale		
	Role	Input		
N	Valid	403		
	Missing	1		
Central Tendency and Dispersion	Mean	1.54		
	Standard Deviation	.792		
	Percentile 25	1.00		
	Percentile 50	1.00		
	Percentile 75	2.00		
Labeled Values	1	No time at all	247	61.1%
	2	A little time (1-2 hrs)	109	27.0%

	3	A moderate amount of time (3-4 hrs)	37	9.2%
	4	A lot of time (5-6 hrs)	7	1.7%
	5	A great deal of time (7+ hrs)	3	0.7%

Lab_TimeLabLead_Day10

		Value	Count	Percent
Standard Attributes	Position	77		
	Label	How much time did you spend with a lab leader other than your primary research mentor, such as a lab supervisor or lab manager in the past 24 hours?		
	Type	Numeric		
	Format	F40		
	Measurement	Scale		
	Role	Input		
N	Valid	299		
	Missing	105		
Central Tendency and Dispersion	Mean	1.41		
	Standard Deviation	.787		

	Percentile 25	1.00		
	Percentile 50	1.00		
	Percentile 75	2.00		
Labeled Values	1	No time at all	214	53.0%
	2	A little time (1-2 hrs)	60	14.9%
	3	A moderate amount of time (3-4 hrs)	14	3.5%
	4	A lot of time (5-6 hrs)	8	2.0%
	5	All day (7+ hrs)	3	0.7%

Lab_TimeLabLead_virtual_Day1

		Value
Standard Attributes	Position	41

	Label	What percent of the time spent with lab leaders other than your primary research mentor was virtual (e.g., video conferencing, such as Zoom or Teams, on the phone, or through email or messaging)? - % of time spent with lab leaders virtually
	Type	Numeric
	Format	F40.2
	Measurement	Scale
	Role	Input
N	Valid	104
	Missing	300
Central Tendency and Dispersion	Mean	34.7115
	Standard Deviation	40.39049
	Percentile 25	2.5000
	Percentile 50	10.0000
	Percentile 75	90.0000

Lab_TimeLabMates_Day10

	Value	Count	Percent

Standard Attributes	Position	78		
	Label	How much time did you spend with other lab mates in the past 24 hours?		
	Type	Numeric		
	Format	F40		
	Measurement	Scale		
	Role	Input		
N	Valid	299		
	Missing	105		
Central Tendency and Dispersion	Mean	2.58		
	Standard Deviation	1.406		
	Percentile 25	1.00		
	Percentile 50	2.00		
	Percentile 75	4.00		
Labeled Values	1	No time at all	97	24.0%
	2	A little time (1-2 hrs)	58	14.4%
	3	A moderate amount of time (3-4 hrs)	55	13.6%
	4	A lot of time (5-6 hrs)	53	13.1%
	5	A great deal of time (7+ hrs)	36	8.9%

Lab_TimeMentor_Day1

		Value	Count	Percent
Standard Attributes	Position	38		
	Label	How much time did you spend with your primary research mentor in the past 24 hours?		
	Type	Numeric		
	Format	F40		
	Measurement	Scale		
	Role	Input		
N	Valid	403		
	Missing	1		
Central Tendency and Dispersion	Mean	1.67		
	Standard Deviation	.717		
	Percentile 25	1.00		
	Percentile 50	2.00		
	Percentile 75	2.00		
Labeled Values	1	No time at all	179	44.3%
	2	A little time (1-2 hrs)	185	45.8%
	3	A moderate amount of time (3-4 hrs)	32	7.9%
	4	A lot of time (5-6 hrs)	5	1.2%

	5	All day (7+ hrs)	2	0.5%

Lab_timeMentor_Day10

		Value	Count	Percent
Standard Attributes	Position	76		
	Label	How much time did you spend with your primary research mentor in the past 24 hours?		
	Type	Numeric		
	Format	F40		
	Measurement	Scale		
	Role	Input		
N	Valid	299		
	Missing	105		
Central Tendency and Dispersion	Mean	1.56		
	Standard Deviation	.723		
	Percentile 25	1.00		
	Percentile 50	1.00		
	Percentile 75	2.00		
Labeled Values	1	No time at all	166	41.1%
	2	A little time (1-2 hrs)	108	26.7%

	3	A moderate amount of time (3-4 hrs)	17	4.2%
	4	A lot of time (5-6 hrs)	8	2.0%
	5	All day (7+ hrs)	0	0.0%

Lab_TimeMentor_virtual_Day1

		Value
Standard Attributes	Position	39
	Label	What percent of the time spent with your primary research mentor was virtual (e.g., video conferencing, such as Zoom or Teams, on the phone, or through email or messaging)? - % of time spent with your primary research mentor virtually
	Type	Numeric
	Format	F40.2
	Measurement	Scale
	Role	Input

N	Valid	193
	Missing	211
Central Tendency and Dispersion	Mean	40.8342
	Standard Deviation	39.39045
	Percentile 25	5.0000
	Percentile 50	24.0000
	Percentile 75	85.0000

Lab_Type_Dry

		Value	Count	Percent
Standard Attributes	Position	33		
	Label	My lab is a Dry lab		
	Type	Numeric		
	Format	F40		
	Measurement	Scale		
	Role	Input		
N	Valid	134		
	Missing	270		
Central Tendency and Dispersion	Mean	1.00		
	Standard Deviation	.000		
	Percentile 25	1.00		
	Percentile 50	1.00		
	Percentile 75	1.00		
Labeled Values	1	Dry lab	134	33.2%

Lab_Type_Field

		Value	Count	Percent
Standard Attributes	Position	35		
	Label	My lab is a Field location		
	Type	Numeric		
	Format	F40		
	Measurement	Scale		
	Role	Input		
N	Valid	6		
	Missing	398		
Central Tendency and Dispersion	Mean	1.00		
	Standard Deviation	.000		
	Percentile 25	1.00		
	Percentile 50	1.00		
	Percentile 75	1.00		
Labeled Values	1	Field location	6	1.5%

Lab_Type_Other

		Value	Count	Percent
Standard Attributes	Position	36		
	Label	My lab is Other		

	Type	Numeric		
	Format	F40		
	Measurement	Scale		
	Role	Input		
N	Valid	17		
	Missing	387		
Central Tendency and Dispersion	Mean	1.00		
	Standard Deviation	.000		
	Percentile 25	1.00		
	Percentile 50	1.00		
	Percentile 75	1.00		
Labeled Values	1	Other (please indicate)	17	4.2%

Lab_Type_other_Text

		Value	Count	Percent
Standard Attributes	Position	37		
	Label	My lab is Other Text		
	Type	String		
	Format	A2000		
	Measurement	Nominal		
	Role	Input		
Valid Values			388	96.0%
	animal facility		1	0.2%

	Animal vivarium and wet lab		1	0.2%
	Behavioral/Clinical		1	0.2%
	clinical research		1	0.2%
	clinical research (human subjects)		1	0.2%
	Clinical research lab		1	0.2%
	epidemiology research group		1	0.2%
	Human lab		1	0.2%
	I do computational work so there is no "lab"		1	0.2%
	I don't work in a lab, just individually with my mentor		1	0.2%
	in my field we don't have labs - Nursing		1	0.2%
	Mouse Behavioral lab		1	0.2%
	Off-site research lab		1	0.2%
	office		1	0.2%
	Office and sometimes going out into community settings like schools or early care centers		1	0.2%
	Research group		1	0.2%

Lab_Type_Virtual

		Value	Count	Percent
Standard Attributes	Position	34		

	Label	My lab is a Virtual lab		
	Type	Numeric		
	Format	F40		
	Measurement	Scale		
	Role	Input		
N	Valid	57		
	Missing	347		
Central Tendency and Dispersion	Mean	1.00		
	Standard Deviation	.000		
	Percentile 25	1.00		
	Percentile 50	1.00		
	Percentile 75	1.00		
Labeled Values	1	Virtual lab	57	14.1%

Lab_Type_Wet

		Value	Count	Percent
Standard Attributes	Position	32		
	Label	My lab is a Wet lab		
	Type	Numeric		
	Format	F40		
	Measurement	Scale		
	Role	Input		
N	Valid	290		

	Missing	114		
Central Tendency and Dispersion	Mean	1.00		
	Standard Deviation	.000		
	Percentile 25	1.00		
	Percentile 50	1.00		
	Percentile 75	1.00		
Labeled Values	1	Wet lab	290	71.8%

LabMates_Day1

		Value	Count	Percent
Standard Attributes	Position	42		
	Label	How much time did you spend with other lab mates in the past 24 hours?		
	Type	Numeric		
	Format	F40		
	Measurement	Scale		
	Role	Input		
N	Valid	403		
	Missing	1		
Central Tendency and Dispersion	Mean	3.03		
	Standard Deviation	1.367		
	Percentile 25	2.00		
	Percentile 50	3.00		

	Percentile 75	4.00		
Labeled Values	1	No time at all	71	17.6%
	2	A little time (1-2 hrs)	76	18.8%
	3	A moderate amount of time (3-4 hrs)	108	26.7%
	4	A lot of time (5-6 hrs)	67	16.6%
	5	A great deal of time (7+ hrs)	81	20.0%

LabMates_virtual_Day1

		Value
Standard Attributes	Position	43
	Label	What percent of the time spent with other lab mates was virtual (e.g., video conferencing, such as Zoom or Teams, on the phone, or through email or messaging)? - % of time spent with other lab mates virtually
	Type	Numeric

	Format	F40.2
	Measurement	Scale
	Role	Input
N	Valid	230
	Missing	174
Central Tendency and Dispersion	Mean	26.2739
	Standard Deviation	32.84220
	Percentile 25	5.0000
	Percentile 50	10.0000
	Percentile 75	31.0000

ProgAtt_1_Day1

		Value	Count	Percent
Standard Attributes	Position	44		
	Label	In the last 24 hours, My commitment to remaining in this program was:		
	Type	Numeric		
	Format	F40		
	Measurement	Scale		
	Role	Input		
N	Valid	402		
	Missing	2		

Central Tendency and Dispersion	Mean	3.13		
	Standard Deviation	.871		
	Percentile 25	3.00		
	Percentile 50	3.00		
	Percentile 75	3.00		
Labeled Values	1	Much lower than normal	19	4.7%
	2	Somewhat lower than normal	38	9.4%
	3	About normal	250	61.9%
	4	Somewhat higher than normal	61	15.1%
	5	Much higher than normal	34	8.4%

ProgAtt_1_Day10

		Value	Count	Percent
Standard Attributes	Position	71		
	Label	Commitment to remain in program		
	Type	Numeric		
	Format	F40		
	Measurement	Scale		
	Role	Input		
N	Valid	299		

	Missing	105		
Central Tendency and Dispersion	Mean	3.20		
	Standard Deviation	.858		
	Percentile 25	3.00		
	Percentile 50	3.00		
	Percentile 75	4.00		
Labeled Values	1	Much lower than normal	13	3.2%
	2	Somewhat lower than normal	21	5.2%
	3	About normal	185	45.8%
	4	Somewhat higher than normal	54	13.4%
	5	Much higher than normal	26	6.4%

ProgAtt_2_Day1

		Value	Count	Percent
Standard Attributes	Position	45		
	Label	In the last 24 hours,My productivity was:		
	Type	Numeric		
	Format	F40		
	Measurement	Scale		
	Role	Input		

N	Valid	403		
	Missing	1		
Central Tendency and Dispersion	Mean	3.06		
	Standard Deviation	.886		
	Percentile 25	3.00		
	Percentile 50	3.00		
	Percentile 75	4.00		
Labeled Values	1	Much lower than normal	13	3.2%
	2	Somewhat lower than normal	86	21.3%
	3	About normal	191	47.3%
	4	Somewhat higher than normal	91	22.5%
	5	Much higher than normal	22	5.4%

ProgAtt_2_Day10

		Value	Count	Percent
Standard Attributes	Position	72		
	Label	Level of productivity		
	Type	Numeric		
	Format	F40		
	Measurement	Scale		

	Role	Input		
N	Valid	299		
	Missing	105		
Central Tendency and Dispersion	Mean	3.03		
	Standard Deviation	1.006		
	Percentile 25	2.00		
	Percentile 50	3.00		
	Percentile 75	4.00		
Labeled Values	1	Much lower than normal	22	5.4%
	2	Somewhat lower than normal	59	14.6%
	3	About normal	129	31.9%
	4	Somewhat higher than normal	67	16.6%
	5	Much higher than normal	22	5.4%

ProgAtt_3_Day1

		Value	Count	Percent
Standard Attributes	Position	46		
	Label	In the last 24 hours, My confidence in my abilities was:		
	Type	Numeric		

	Format	F40		
	Measurement	Scale		
	Role	Input		
N	Valid	403		
	Missing	1		
Central Tendency and Dispersion	Mean	3.17		
	Standard Deviation	.858		
	Percentile 25	3.00		
	Percentile 50	3.00		
	Percentile 75	4.00		
Labeled Values	1	Much lower than normal	16	4.0%
	2	Somewhat lower than normal	50	12.4%
	3	About normal	210	52.0%
	4	Somewhat higher than normal	105	26.0%
	5	Much higher than normal	22	5.4%

ProgAtt_3_Day10

		Value	Count	Percent
Standard Attributes	Position	73		
	Label	Confidence in ability		

	Type	Numeric		
	Format	F40		
	Measurement	Scale		
	Role	Input		
N	Valid	299		
	Missing	105		
Central Tendency and Dispersion	Mean	3.16		
	Standard Deviation	.814		
	Percentile 25	3.00		
	Percentile 50	3.00		
	Percentile 75	4.00		
Labeled Values	1	Much lower than normal	8	2.0%
	2	Somewhat lower than normal	40	9.9%
	3	About normal	163	40.3%
	4	Somewhat higher than normal	73	18.1%
	5	Much higher than normal	15	3.7%

ProgAtt_4_Day1

		Value	Count	Percent
Standard Attributes	Position	47		

	Label	In the last 24 hours,My satisfaction in my graduate/post-doc program was:		
	Type	Numeric		
	Format	F40		
	Measurement	Scale		
	Role	Input		
N	Valid	403		
	Missing	1		
Central Tendency and Dispersion	Mean	2.92		
	Standard Deviation	.875		
	Percentile 25	3.00		
	Percentile 50	3.00		
	Percentile 75	3.00		
Labeled Values	1	Much lower than normal	29	7.2%
	2	Somewhat lower than normal	69	17.1%
	3	About normal	225	55.7%
	4	Somewhat higher than normal	65	16.1%
	5	Much higher than normal	15	3.7%

ProgAtt_4_Day10

		Value	Count	Percent
Standard Attributes	Position	74		
	Label	Satisfaction in training program		
	Type	Numeric		
	Format	F40		
	Measurement	Scale		
	Role	Input		
N	Valid	299		
	Missing	105		
Central Tendency and Dispersion	Mean	3.00		
	Standard Deviation	.837		
	Percentile 25	3.00		
	Percentile 50	3.00		
	Percentile 75	3.00		
Labeled Values	1	Much lower than normal	21	5.2%
	2	Somewhat lower than normal	31	7.7%
	3	About normal	183	45.3%
	4	Somewhat higher than normal	54	13.4%
	5	Much higher than normal	10	2.5%

RaceMT_1_Day1

		Value	Count	Percent
Standard Attributes	Position	56		
	Label	In the past 24 hours, Someone in my lab engaged in racist behavior toward me or others.		
	Type	Numeric		
	Format	F40		
	Measurement	Scale		
	Role	Input		
N	Valid	403		
	Missing	1		
Central Tendency and Dispersion	Mean	.04		
	Standard Deviation	.219		
	Percentile 25	.00		
	Percentile 50	.00		
	Percentile 75	.00		
Labeled Values	0	Definitely not	389	96.3%
	1	Possibly, but not sure	12	3.0%
	2	Yes, definitely	2	0.5%

RaceMT_1_Day10

		Value	Count	Percent
Standard Attributes	Position	92		
	Label	Race-based Sex Harassment Item 1: Someone in my lab engaged in racist behavior toward me or others		
	Type	Numeric		
	Format	F40		
	Measurement	Scale		
	Role	Input		
N	Valid	299		
	Missing	105		
Central Tendency and Dispersion	Mean	.04		
	Standard Deviation	.220		
	Percentile 25	.00		
	Percentile 50	.00		
	Percentile 75	.00		
Labeled Values	0	Definitely not	287	71.0%
	1	Possibly, but not sure	11	2.7%
	2	Yes, definitely	1	0.2%

RaceMT_2_Day1

		Value	Count	Percent
Standard Attributes	Position	57		
	Label	In the past 24 hours, Someone in my lab engaged in racially crude behavior toward me or others.		
	Type	Numeric		
	Format	F40		
	Measurement	Scale		
	Role	Input		
N	Valid	403		
	Missing	1		
Central Tendency and Dispersion	Mean	.03		
	Standard Deviation	.197		
	Percentile 25	.00		
	Percentile 50	.00		
	Percentile 75	.00		
Labeled Values	0	Definitely not	393	97.3%
	1	Possibly, but not sure	8	2.0%
	2	Yes, definitely	2	0.5%

RaceMT_2_Day10

		Value	Count	Percent

Standard Attributes	Position	93		
	Label	Race-based Sex Harassment Item 2: Someone in my lab engaged in racially crude behavior toward me or otehrs		
	Type	Numeric		
	Format	F40		
	Measurement	Scale		
	Role	Input		
N	Valid	299		
	Missing	105		
Central Tendency and Dispersion	Mean	.04		
	Standard Deviation	.235		
	Percentile 25	.00		
	Percentile 50	.00		
	Percentile 75	.00		
Labeled Values	0	Definitely not	288	71.3%
	1	Possibly, but not sure	9	2.2%
	2	Yes, definitely	2	0.5%

RaceMT_3_Day1

		Value	Count	Percent

Standard Attributes	Position	58		
	Label	In the past 24 hours, Someone in my lab made assumptions that I or other minorities were inferior.		
	Type	Numeric		
	Format	F40		
	Measurement	Scale		
	Role	Input		
N	Valid	403		
	Missing	1		
Central Tendency and Dispersion	Mean	.06		
	Standard Deviation	.280		
	Percentile 25	.00		
	Percentile 50	.00		
	Percentile 75	.00		
Labeled Values	0	Definitely not	382	94.6%
	1	Possibly, but not sure	17	4.2%
	2	Yes, definitely	4	1.0%

RaceMT_3_Day10

		Value	Count	Percent
Standard Attributes	Position	94		

	Label	Race-based Microaggressions Item 1: Someone in my lab made assumptions that I or other minorities were inferior		
	Type	Numeric		
	Format	F40		
	Measurement	Scale		
	Role	Input		
N	Valid	299		
	Missing	105		
Central Tendency and Dispersion	Mean	.05		
	Standard Deviation	.233		
	Percentile 25	.00		
	Percentile 50	.00		
	Percentile 75	.00		
Labeled Values	0	Definitely not	285	70.5%
	1	Possibly, but not sure	13	3.2%
	2	Yes, definitely	1	0.2%

RaceMT_4_Day1

		Value	Count	Percent
Standard Attributes	Position	59		

	Label	In the past 24 hours, Someone in my lab treated me or other minorities as a second-class citizen.		
	Type	Numeric		
	Format	F40		
	Measurement	Scale		
	Role	Input		
N	Valid	403		
	Missing	1		
Central Tendency and Dispersion	Mean	.06		
	Standard Deviation	.293		
	Percentile 25	.00		
	Percentile 50	.00		
	Percentile 75	.00		
Labeled Values	0	Definitely not	385	95.3%
	1	Possibly, but not sure	12	3.0%
	2	Yes, definitely	6	1.5%

RaceMT_4_Day10

		Value	Count	Percent
Standard Attributes	Position	95		

	Label	Race-based Microaggressions Item 2: Someone in my lab treated me or other minorities as a second-class citizen		
	Type	Numeric		
	Format	F40		
	Measurement	Scale		
	Role	Input		
N	Valid	299		
	Missing	105		
Central Tendency and Dispersion	Mean	.04		
	Standard Deviation	.204		
	Percentile 25	.00		
	Percentile 50	.00		
	Percentile 75	.00		
Labeled Values	0	Definitely not	286	70.8%
	1	Possibly, but not sure	13	3.2%
	2	Yes, definitely	0	0.0%

RaceMT_5_Day1

		Value	Count	Percent

Standard Attributes	Position	60		
	Label	In the past 24 hours, Someone in my lab invalidated me or other minorities' experiences as a person of color.		
	Type	Numeric		
	Format	F40		
	Measurement	Scale		
	Role	Input		
N	Valid	403		
	Missing	1		
Central Tendency and Dispersion	Mean	.05		
	Standard Deviation	.268		
	Percentile 25	.00		
	Percentile 50	.00		
	Percentile 75	.00		
Labeled Values	0	Definitely not	385	95.3%
	1	Possibly, but not sure	14	3.5%
	2	Yes, definitely	4	1.0%

RaceMT_5_Day10

		Value	Count	Percent

Standard Attributes	Position	96		
	Label	Race-based Microaggressions Item 3: Someone in my lab invalidated me or other minorities experiences as a person of color		
	Type	Numeric		
	Format	F40		
	Measurement	Scale		
	Role	Input		
N	Valid	299		
	Missing	105		
Central Tendency and Dispersion	Mean	.04		
	Standard Deviation	.213		
	Percentile 25	.00		
	Percentile 50	.00		
	Percentile 75	.00		
Labeled Values	0	Definitely not	288	71.3%
	1	Possibly, but not sure	10	2.5%
	2	Yes, definitely	1	0.2%

RaceMT_6_Day1

		Value	Count	Percent
Standard Attributes	Position	61		
	Label	In the past 24 hours, Someone in my lab was subtly aggressive toward me or other minorities.		
	Type	Numeric		
	Format	F40		
	Measurement	Scale		
	Role	Input		
N	Valid	403		
	Missing	1		
Central Tendency and Dispersion	Mean	.10		
	Standard Deviation	.373		
	Percentile 25	.00		
	Percentile 50	.00		
	Percentile 75	.00		
Labeled Values	0	Definitely not	373	92.3%
	1	Possibly, but not sure	20	5.0%
	2	Yes, definitely	10	2.5%

RaceMT_6_Day10

		Value	Count	Percent

Standard Attributes	Position	97		
	Label	Race-based Microaggressions Item 4: Someone in my lab was subtly aggressive toward me or other minorities		
	Type	Numeric		
	Format	F40		
	Measurement	Scale		
	Role	Input		
N	Valid	299		
	Missing	105		
Central Tendency and Dispersion	Mean	.06		
	Standard Deviation	.246		
	Percentile 25	.00		
	Percentile 50	.00		
	Percentile 75	.00		
Labeled Values	0	Definitely not	283	70.0%
	1	Possibly, but not sure	15	3.7%
	2	Yes, definitely	1	0.2%

RaceMT_7_Day1

		Value	Count	Percent

Standard Attributes	Position	62		
	Label	In the past 24 hours, Someone in my lab ignored me or other minorities or made us feel invisible.		
	Type	Numeric		
	Format	F40		
	Measurement	Scale		
	Role	Input		
N	Valid	403		
	Missing	1		
Central Tendency and Dispersion	Mean	.10		
	Standard Deviation	.367		
	Percentile 25	.00		
	Percentile 50	.00		
	Percentile 75	.00		
Labeled Values	0	Definitely not	372	92.1%
	1	Possibly, but not sure	22	5.4%
	2	Yes, definitely	9	2.2%

RaceMT_7_Day10

		Value	Count	Percent
Standard Attributes	Position	98		

	Label	Race-based Microagressions Item 5: Someone in my lab ignored me or other minorities or made us feel invisible		
	Type	Numeric		
	Format	F40		
	Measurement	Scale		
	Role	Input		
N	Valid	299		
	Missing	105		
Central Tendency and Dispersion	Mean	.06		
	Standard Deviation	.246		
	Percentile 25	.00		
	Percentile 50	.00		
	Percentile 75	.00		
Labeled Values	0	Definitely not	283	70.0%
	1	Possibly, but not sure	15	3.7%
	2	Yes, definitely	1	0.2%

S_HARM_Day1

		Value	Count	Percent
Standard Attributes	Position	68		

	Label	Sum of SexHarass, RaceMT, and Incivility, Day 1		
	Type	Numeric		
	Format	F8.2		
	Measurement	Nominal		
	Role	Input		
Valid Values	.00		274	67.8%
	1.00		41	10.1%
	2.00		31	7.7%
	3.00		16	4.0%
	4.00		14	3.5%
	5.00		3	0.7%
	6.00		4	1.0%
	7.00		2	0.5%
	8.00		2	0.5%
	9.00		5	1.2%
	10.00		1	0.2%
	12.00		1	0.2%
	13.00		2	0.5%
	14.00		1	0.2%
	15.00		1	0.2%
	16.00		1	0.2%
	17.00		2	0.5%
	18.00		1	0.2%

	19.00		1	0.2%
Missing Values	System		1	0.2%

S_HARM_Day10

		Value	Count	Percent
Standard Attributes	Position	102		
	Label	Sum of SexHarass, RaceMT, and Incivility Day 10		
	Type	Numeric		
	Format	F8.2		
	Measurement	Nominal		
	Role	Input		
Valid Values	.00		234	57.9%
	1.00		23	5.7%
	2.00		16	4.0%
	3.00		5	1.2%
	4.00		6	1.5%
	6.00		7	1.7%
	8.00		1	0.2%
	9.00		3	0.7%
	10.00		3	0.7%
	14.00		1	0.2%
Missing Values	System		105	26.0%

S_INCIV_Day10

		Value	Count	Percent
Standard Attributes	Position	100		
	Label	sum of incivility items on Day 10		
	Type	Numeric		
	Format	F8.2		
	Measurement	Nominal		
	Role	Input		
Valid Values	.00		240	59.4%
	1.00		22	5.4%
	2.00		14	3.5%
	3.00		10	2.5%
	4.00		5	1.2%
	5.00		1	0.2%
	6.00		7	1.7%
Missing Values	System		105	26.0%

S_INCV_Day1

		Value	Count	Percent
Standard Attributes	Position	66		
	Label	Sum of incivility experienced on Day 1		

	Type	Numeric		
	Format	F8.2		
	Measurement	Nominal		
	Role	Input		
Valid Values	.00		296	73.3%
	1.00		38	9.4%
	2.00		35	8.7%
	3.00		10	2.5%
	4.00		23	5.7%
	9.00		1	0.2%
Missing Values	System		1	0.2%

S_Micro_Day1

		Value	Count	Percent
Standard Attributes	Position	70		
	Label	COMPUTE S_Micro_Day1=sum(RaceMicro_1_Day1,RaceMicro_2_Day1,RaceMicro_3_Day1,RaceMicro_4_Day1,RaceMicro_5_Day1)		
	Type	Numeric		
	Format	F8.2		
	Measurement	Nominal		

	Role	Input		
Valid Values	.00		356	88.1%
	1.00		13	3.2%
	2.00		15	3.7%
	3.00		1	0.2%
	4.00		4	1.0%
	5.00		8	2.0%
	6.00		1	0.2%
	7.00		1	0.2%
	8.00		1	0.2%
	9.00		2	0.5%
	10.00		1	0.2%
Missing Values	System		1	0.2%

S_ProgAtt_Day1

		Value
Standard Attributes	Position	48
	Label	Average of four program attitude items experienced on Day 1
	Type	Numeric
	Format	F8.2
	Measurement	Scale
	Role	Input

N	Valid	403
	Missing	1
Central Tendency and Dispersion	Mean	3.0697
	Standard Deviation	.65294
	Percentile 25	2.7500
	Percentile 50	3.0000
	Percentile 75	3.5000

S_ProgAtt_Day10

		Value
Standard Attributes	Position	75
	Label	COMPUTE S_ProgAtt_Day10= MEAN(ProgAtt_1_Day10,ProgAtt_2_Day10,ProgAtt_3_Day10,ProgAtt_4_Day10)
	Type	Numeric
	Format	F8.2
	Measurement	Scale
	Role	Input
N	Valid	299
	Missing	105
Central Tendency and Dispersion	Mean	3.0962
	Standard Deviation	.64875

	Percentile 25	2.7500
	Percentile 50	3.0000
	Percentile 75	3.5000

S_RaceMT_Day1

		Value	Count	Percent
Standard Attributes	Position	67		
	Label	COMPUTE S_RaceMT_Day1=sum(S_RH_Day1,S_Micro_Day1)		
	Type	Numeric		
	Format	F8.2		
	Measurement	Nominal		
	Role	Input		
Valid Values	.00		355	87.9%
	1.00		13	3.2%
	2.00		13	3.2%
	3.00		2	0.5%
	4.00		5	1.2%
	5.00		3	0.7%
	6.00		2	0.5%
	7.00		4	1.0%
	8.00		1	0.2%
	9.00		2	0.5%

	10.00		1	0.2%
	11.00		1	0.2%
	12.00		1	0.2%
Missing Values	System		1	0.2%

S_RaceMT_Day10

		Value	Count	Percent
Standard Attributes	Position	101		
	Label	COMPUTE S_RaceMT_Day10= sum(S_RH_Day10, S_Micro_Day10)		
	Type	Numeric		
	Format	F8.2		
	Measurement	Nominal		
	Role	Input		
Valid Values	.00		273	67.6%
	1.00		9	2.2%
	2.00		5	1.2%
	4.00		5	1.2%
	5.00		3	0.7%
	6.00		1	0.2%
	7.00		2	0.5%
	10.00		1	0.2%
Missing Values	System		105	26.0%

S_RH_Day1

		Value	Count	Percent
Standard Attributes	Position	69		
	Label	COMPUTE S_RH_Day1=sum(RaceHarass_L_1_Day1,RaceHarass_L_2_Day1)		
	Type	Numeric		
	Format	F8.2		
	Measurement	Nominal		
	Role	Input		
Valid Values	.00		389	96.3%
	1.00		4	1.0%
	2.00		8	2.0%
	4.00		2	0.5%
Missing Values	System		1	0.2%

S_SexHarass_Day1

		Value	Count	Percent
Standard Attributes	Position	65		
	Label	Sum of SH experienced on Day 1		

	Type	Numeric		
	Format	F8.2		
	Measurement	Nominal		
	Role	Input		
Valid Values	.00		367	90.8%
	1.00		16	4.0%
	2.00		14	3.5%
	3.00		3	0.7%
	4.00		2	0.5%
	6.00		1	0.2%
Missing Values	System		1	0.2%

S_SexHarass_Day10

		Value	Count	Percent
Standard Attributes	Position	99		
	Label	the sum of the four SexHarass items on Day 10		
	Type	Numeric		
	Format	F8.2		
	Measurement	Nominal		
	Role	Input		
Valid Values	.00		278	68.8%
	1.00		12	3.0%

	2.00		3	0.7%
	4.00		6	1.5%
Missing Values	System		105	26.0%

SexHarass_1_Day1

		Value	Count	Percent
Standard Attributes	Position	49		
	Label	In the past 24 hours, Someone in my lab engaged in sexist behavior toward me or others.		
	Type	Numeric		
	Format	F40		
	Measurement	Scale		
	Role	Input		
N	Valid	403		
	Missing	1		
Central Tendency and Dispersion	Mean	.09		
	Standard Deviation	.354		
	Percentile 25	.00		
	Percentile 50	.00		
	Percentile 75	.00		
Labeled Values	0	Definitely not	373	92.3%

	1	Possibly, but not sure	22	5.4%
	2	Yes, definitely	8	2.0%

SexHarass_1_Day10

		Value	Count	Percent
Standard Attributes	Position	79		
	Label	SexHarass Item 1: Someone in my lab engaged in sexist behavior toward me or others		
	Type	Numeric		
	Format	F40		
	Measurement	Scale		
	Role	Input		
N	Valid	299		
	Missing	105		
Central Tendency and Dispersion	Mean	.06		
	Standard Deviation	.294		
	Percentile 25	.00		
	Percentile 50	.00		
	Percentile 75	.00		
Labeled Values	0	Definitely not	284	70.3%
	1	Possibly, but not sure	11	2.7%

	2	Yes, definitely	4	1.0%

SexHarass_1_targetgender_Day10

		Value	Count	Percent
Standard Attributes	Position	83		
	Label	Target of behavior: Me		
	Type	Numeric		
	Format	F40		
	Measurement	Scale		
	Role	Input		
N	Valid	299		
	Missing	105		
Central Tendency and Dispersion	Mean	.04		
	Standard Deviation	.197		
	Percentile 25	.00		
	Percentile 50	.00		
	Percentile 75	.00		
Labeled Values	1	Me	12	3.0%

SexHarass_2_Day1

		Value	Count	Percent
Standard Attributes	Position	50		

	Label	In the past 24 hours, Someone in my lab engaged in sexually crude behavior toward me or others.		
	Type	Numeric		
	Format	F40		
	Measurement	Scale		
	Role	Input		
N	Valid	403		
	Missing	1		
Central Tendency and Dispersion	Mean	.03		
	Standard Deviation	.197		
	Percentile 25	.00		
	Percentile 50	.00		
	Percentile 75	.00		
Labeled Values	0	Definitely not	393	97.3%
	1	Possibly, but not sure	8	2.0%
	2	Yes, definitely	2	0.5%

SexHarass_2_Day10

		Value	Count	Percent
Standard Attributes	Position	80		

	Label	SexHarass Item 2: Someone in my lab engaged in sexually crude behavior toward me or others		
	Type	Numeric		
	Format	F40		
	Measurement	Scale		
	Role	Input		
N	Valid	299		
	Missing	105		
Central Tendency and Dispersion	Mean	.03		
	Standard Deviation	.229		
	Percentile 25	.00		
	Percentile 50	.00		
	Percentile 75	.00		
Labeled Values	0	Definitely not	292	72.3%
	1	Possibly, but not sure	4	1.0%
	2	Yes, definitely	3	0.7%

SexHarass_2_targetgender_Day10

		Value	Count	Percent
Standard Attributes	Position	84		

	Label	Target of behavior: Other women		
	Type	Numeric		
	Format	F40		
	Measurement	Scale		
	Role	Input		
N	Valid	299		
	Missing	105		
Central Tendency and Dispersion	Mean	.03		
	Standard Deviation	.162		
	Percentile 25	.00		
	Percentile 50	.00		
	Percentile 75	.00		
Labeled Values	1	Other women	8	2.0%

SexHarass_3_Day1

		Value	Count	Percent
Standard Attributes	Position	51		
	Label	In the past 24 hours, Someone in my lab gave me or others unwanted sexual attention.		
	Type	Numeric		
	Format	F40		

	Measurement	Scale		
	Role	Input		
N	Valid	403		
	Missing	1		
Central Tendency and Dispersion	Mean	.04		
	Standard Deviation	.202		
	Percentile 25	.00		
	Percentile 50	.00		
	Percentile 75	.00		
Labeled Values	0	Definitely not	389	96.3%
	1	Possibly, but not sure	13	3.2%
	2	Yes, definitely	1	0.2%

SexHarass_3_Day10

		Value	Count	Percent
Standard Attributes	Position	81		
	Label	SexHarass Item 3: Someone in my lab gave me or others unwanted sexual attention		
	Type	Numeric		
	Format	F40		
	Measurement	Scale		

	Role	Input		
N	Valid	299		
	Missing	105		
Central Tendency and Dispersion	Mean	.03		
	Standard Deviation	.214		
	Percentile 25	.00		
	Percentile 50	.00		
	Percentile 75	.00		
Labeled Values	0	Definitely not	291	72.0%
	1	Possibly, but not sure	6	1.5%
	2	Yes, definitely	2	0.5%

SexHarass_3_targetgender_Day10

		Value	Count	Percent
Standard Attributes	Position	85		
	Label	Target of behavior: Other men		
	Type	Numeric		
	Format	F40		
	Measurement	Scale		
	Role	Input		
N	Valid	299		
	Missing	105		

Central Tendency and Dispersion	Mean	.01		
	Standard Deviation	.100		
	Percentile 25	.00		
	Percentile 50	.00		
	Percentile 75	.00		
Labeled Values	1	Other men	3	0.7%

SexHarass_4_Day1

		Value	Count	Percent
Standard Attributes	Position	52		
	Label	In tn the past 24 hours, Someone in my labimplied that I or others would be treated differently if we cooperated sexually with them.		
	Type	Numeric		
	Format	F40		
	Measurement	Scale		
	Role	Input		
N	Valid	403		
	Missing	1		
Central Tendency and Dispersion	Mean	.00		
	Standard Deviation	.070		

	Percentile 25	.00		
	Percentile 50	.00		
	Percentile 75	.00		
Labeled Values	0	Definitely not	401	99.3%
	1	Possibly, but not sure	2	0.5%
	2	Yes, definitely	0	0.0%

SexHarass_4_Day10

		Value	Count	Percent
Standard Attributes	Position	82		
	Label	SexHarass Item 4: Someone in my lab implied that I or others would be treated differently if we cooperated sexually with them		
	Type	Numeric		
	Format	F40		
	Measurement	Scale		
	Role	Input		
N	Valid	299		
	Missing	105		
Central Tendency and Dispersion	Mean	.01		
	Standard Deviation	.100		

	Percentile 25	.00		
	Percentile 50	.00		
	Percentile 75	.00		
Labeled Values	0	Definitely not	296	73.3%
	1	Possibly, but not sure	3	0.7%
	2	Yes, definitely	0	0.0%

SexHarass_4_targetgender_Day10

		Value	Count	Percent
Standard Attributes	Position	86		
	Label	Other people or persons with a different gender identity		
	Type	Numeric		
	Format	F40		
	Measurement	Scale		
	Role	Input		
N	Valid	299		
	Missing	105		
Central Tendency and Dispersion	Mean	.01		
	Standard Deviation	.115		
	Percentile 25	.00		
	Percentile 50	.00		

	Percentile 75	.00		
Labeled Values	1	Other people/person with a different gender identity	4	1.0%
