# Supplementary material for: Prevalence and Impact of Single-Day Events of Sexual Harassment, Racial Mistreatment, and Incivility on Biomedical Health Trainees: A Mixed-Methods Study
Source: Behav Sci (Basel). 2026 Mar 6;16(3):380. doi: 10.3390/bs16030380 (PMC13024630; doi:10.3390/bs16030380)
Supplement: Supplementary file 1 [file behavsci-16-00380-s001.zip › Supplementary Files/Study 1 Day 10 Questionnaire.pdf]

Introduction

Thank you for agreeing to participate in this study. Please complete the survey in a place where you have privacy. Your responses are confidential. Please answer truthfully. The survey should take approximately 10 minutes to complete.

Work Outcomes

The following series of questions ask you to indicate your attitudes and experiences in the past 24 hours so that we can gain an assessment of your most recent feelings and experiences.

Please complete the following sentences based on how you have felt over the **past 24 hours**.

	Much lower than normal	Somewhat lower than normal	About normal	Somewhat higher than normal	Much higher than normal
My commitment to remaining in this program was:	○	○	○	○	○
My productivity was:	○	○	○	○	○
My confidence in my abilities was:	○	○	○	○	○
My satisfaction in my graduate/post-doc program was:	○	○	○	○	○

Frequency of Daily Interactions

How much time did you spend with your lab PI in the past 24 hours?

No time at all | A little time | A moderate | A lot of time | All day

amount of time

How much time did you spend with a lab leader other than your PI, such as a lab supervisor or lab manager in the past 24 hours?

No time at all	A little time	A moderate amount of time	A lot of time	All day
○	○	○	○	○

How much time did you spend with other lab mates in the past 24 hours?

No time at all	A little time	A moderate amount of time	A lot of time	A great deal of time
○	○	○	○	○

What percent of the time that you spent with your lab PI/mentor, other lab leader and lab mates was virtual (e.g., video conferencing, such as Zoom or Teams, on the phone, or through email or messaging)?

	0	10	20	30	40	50	60	70	80	90	100
Lab PI/Mentor											
Other lab leader											
Lab mates											

Experiences Introduction

For the following set of questions, you will be asked to rate positive and negative experiences you may have had in the past 24 hours in your lab. Please interpret "lab" to mean any setting where you were working on research. Please

interpret "someone in my lab" to mean any person you may have worked with or been in contact with where you were working on research. This could be your primary mentor/PI, another mentor, a lab supervisor, staff person, technician, other students or fellows, or anyone else.

Allyship Experiences

Please indicate the extent to which you experienced the following events in the past 24 hours.

	Not at all	Somewhat	Yes, definitely
Someone in my lab supported me in a significant way.	◯	◯	◯
Someone in my lab showed that they were an ally toward me.	◯	◯	◯
Someone in my lab stepped in to intervene against mistreatment toward me or other lab mates.	◯	◯	◯
Someone in my lab mentored me.	◯	◯	◯

Unwanted Sexist, Sexual, Racist, or Uncivil Experiences

Please indicate the extent to which you experienced the following events in the past 24 hours.

	Not at all	Somewhat	Yes, definitely

	Not at all	Somewhat	Yes, definitely
Someone in my lab engaged in sexist behavior toward me or others.	○	○	○
Someone in my lab engaged in sexually crude behavior toward me or others.	○	○	○
Someone in my lab gave me or others unwanted sexual attention.	○	○	○
Someone in my lab implied that I or others would be treated differently if we cooperated sexually with them.	○	○	○

Who was this behavior directed toward? (check all that apply)

- ☐ Me
- ☐ Other women
- ☐ Other men
- ☐ Other people/person with a different gender identity

Please indicate the extent to which you experienced the following events in the past 24 hours.

	Not at all	Somewhat	Yes, definitely
Someone in my lab put me down or was condescending to me or others.	○	○	○

	Not at all	Somewhat	Yes, definitely
Someone in my lab paid little attention to my or others' opinions.	◯	◯	◯
Someone in my lab addressed me or others in unprofessional terms either publicly or in private.	◯	◯	◯

Who was this behavior directed toward? (check all that apply)

☐ Me

☐ Other

Please indicate the extent to which you experienced the following events in the past 24 hours.

	Not at all	Somewhat	Yes, definitely
Someone in my lab engaged in racist behavior toward me or others.	◯	◯	◯
Someone in my lab engaged in racially crude behavior toward me or others.	◯	◯	◯

Who was this behavior directed toward? (check all that apply)

☐ Me

☐ Others

Please indicate the extent to which you experienced the following events in the past 24 hours.

	Not at all	Somewhat	Yes, definitely
Someone in my lab made assumptions that I or other minorities were inferior.	○	○	○
Someone in my lab treated me or other minorities as a second-class citizen.	○	○	○
Someone in my lab invalidated me or other minorities' experiences as a person of color.	○	○	○
Someone in my lab was subtly aggressive toward me or other minorities.	○	○	○
Someone in my lab ignored me or other minorities or made us feel invisible.	○	○	○

Who was this behavior directed toward? (check all that apply)

- ☐ Me
- ☐ Other people with minoritized identities
- ☐ Other people who do not have minoritized identities

Perpetrator

Was the person or persons who did this:

	Yes	No
The PI or lead professor of your lab?	◯	◯
Another lab leader, such as a lab manager or senior lab member?	◯	◯
Another lab member?	◯	◯
One or more men?	◯	◯
One or more women?	◯	◯
One or more gender nonbinary people?	◯	◯
A combination of men, women, or gender nonbinary people?	◯	◯

If you need assistance with the treatment you experienced today, please contact your institution's Equal Employment office, Title IX coordinator, or someone in Student Affairs. For general assistance, contact the National Sexual Assault Hotline: 1-800-656-4673. Live chat is available at rainn.org.

ExperiencesText_T2

If you would like, please use this space to tell us more about your experiences working in this lab.

ShareStory

Thank you for responding to this survey. Your responses have been recorded.

You indicated that you have experienced harassment or racial mistreatment. We are seeking to interview individuals who have experienced sexual harassment or racial mistreatment in the past and are willing to talk about their story to a clinician-in-training. If selected, your interview will be re-enacted by an actor and videotaped. An edited version of the videotape will then be used in an experimental training program designed to mitigate sexual harassment and racial mistreatment, as well as in future research on these videos.

The information you provide in your interview (if selected) will remain confidential. The videotaped re-enactment will be made in such a way that you cannot be identified. You will have the opportunity to review and approve the videotape before it is used for training or research studies.

Individuals who are selected for the interview and who fully participate in the interview process will be provided an honorarium of $200.

Are you interested in being contacted by a member of the research team about being interviewed about your experience(s) of sexual harassment?

Note: Not all individuals who express interest or who are initially contacted may be selected to be interviewed).

- ◯ Yes, I am interested
- ◯ No, I am not interested

What are good ways to reach you? Please check all that apply and provide your contact information. This information will be kept confidential and will not be connected to your responses on this survey.

- ☐ ______ Phone (My phone number is:)
- ☐ ______ Email (My preferred email address is:)
- ☐ ______ Text message (My cell number is:)
- ☐ ______ Other

Thank you! A member of the research team will be in touch.

Powered by Qualtrics
