# Supplementary material for: Prevalence and Impact of Single-Day Events of Sexual Harassment, Racial Mistreatment, and Incivility on Biomedical Health Trainees: A Mixed-Methods Study
Source: Behav Sci (Basel). 2026 Mar 6;16(3):380. doi: 10.3390/bs16030380 (PMC13024630; doi:10.3390/bs16030380)
Supplement: Supplementary file 1 [file behavsci-16-00380-s001.zip › Supplementary Files/Study 2 Brian Interview Transcript.pdf]

Brian Interview Transcript

Interviewer: Hi Brian. Can you tell me how this all started? And how did you come to know, Dan?

Brian: So, I got into graduate school, in micro and Dan and I were in the same cohort. We rotated into Dr. Thompson's lab. She was an intense micro-manager and what not, but I thought, maybe I'll join that lab. Dan kind of pressured me to join the lab because he was worried about her tendencies to micro-manage. But I thought I could deal with it so I ended up joining the lab because I thought it would give me the best opportunity to grow. The questions were interesting.

I noticed he was very insecure. He always wanted to show that he was the smartest person in the room. It's like it's constantly this dominance thing. He would start to make lewd comments about me. I thought he was joking. He would say, "oh you look like a snack today," or like "I want to fuck you," or stuff like that. And I thought "oh, he's an abrasive guy. He has a crude sense of humor." But I don't want to cause tension in the lab. I don't want to cause issues with my advisor, either. So, I kinda normalized his behavior.

Dr. Thompson fostered that kind of environment. She would actively show favoritism and it would change from week to week. It's very hard to talk to Dr. Thompson about any other issues including science because everything with her is always black and white So, that's how the harassment accumulated over time, because it was, like, a slow escalation.

Interviewer: Could you talk a little bit more about how the harassment escalated over time?

Brian: So, in the first 8-12 months, he would always try to find his limit, like how far could could push me. He would either make those lewd comments or just say some crazy shit, just to get a reaction from me.

In my second year, he would push the boundaries, like put me down in front of our friends. But I would laugh it off. I didn't want to be the person who causes the scene. By my third and fourth year is when, like, the sexual harassment started. Halfway through my third or fourth year he began to touch me, like on my shoulder at first. He would come up to me and grab my ass. He'd hover over me. I could feel him breathing on me. The sexual harassment didn't happen in front of Dr. Thompson, but he would make a weird comment about how I looked in front of her, and her response would just be to laugh it off. She kind of created

this, toxic, competitive environment.

One day we had a department social. I was completely uncomfortable to be around Dan, so I was off on the side of the room. I bent over to pick up a ping pong ball that rolled near me and he grabbed my ass in front of everyone. No one said anything. I had kind of hit my breaking point. This was taking a severe toll on me mentally. And I thought, “I'm not going to be able to finish my PhD if things remain the same.”

So, it kind of went from there to where he got more bold. He would put his crotch in front of my face and…sorry for a lack of better words. he would grab my crotch. It got to the point where he was stalking me. He wanted to know where I was going, what meetings I had. If I grabbed my coat, he would stop me, “where are you going” and “why wasn't I invited.” I was in survival mode. I just wanted to get through another year and a half, and I'd be done. It was clear I was trying to avoid him. I would tell him to stop and push him away.

I eventually reported him to Dr. Thompson. I told her not to report it but she said she would have to report it. It felt like everything went crazy from there. Because we were students, the Office of Equal Opportunity didn't want to treat Dan like an employee where he would be banned from campus. They wanted to silence the problem. We had to keep a Google calendar so that we wouldn't be on campus at the same time on weekends when we needed to be in the lab. We couldn't share the same bathroom. I was no longer allowed to talk about it anymore, because it would be considered bullying or ganging up on him. I went to Dr. Thompson and said this is ridiculous. Could you relocate him. He agreed to relocate so that he could finish up his work. But there was no punishment for him. His PhD was expedited. He got his PhD 9-12 months earlier than he would have and he got a job with a biotech with no history following him. I told Dr. Thompson that it was insane that he got his PhD faster because he sexually harassed me. She said that he deserved his PhD because he was a good student. She invalidated how I felt about the sexual harassment and the PhD process. It took the wind out of my sails to the point where I was barely functioning.

Interviewer: I‘m sure that in retelling it at this moment it brings up some emotions. What were some of the emotions that you were feeling and how did they change over time. How did the experience affect how you think about yourself?

Brian: I guess at the start, I felt uncomfortable, but I could still function. He would grab my shoulder, but I thought that's just the way you are, and I think that's fine, I mean it's not fine, but I was trying to normalize it. I wanted to leave my frustrations at the door. When I got home, I didn't want the problems to bleed over with my partner, my friendships, or my

home life. People weren't seeing it and I thought "it must be me." So, I thought it was my fault; I'm the one making it hard for people to get along with me. It slowly became harder and harder to function and I didn't realize it for a while. I started to break down in tears without having any explanation. When he would touch me, a shock went through me. I lost interest in science. I felt as if I were being gaslit. I didn't know what was real anymore. I was starting to isolate myself. I started to consider alternatives. I thought about quitting graduate school, which felt like a loss.

When I first started graduate school, I felt a lot more confident. I was joyful and laughed a lot. Now I feel like a shell of myself. I've been diagnosed with PTSD because of this experience. I feel very low self-confidence. I beat myself up a lot now with everything. It's just kind of shitty. With my partner, it did take a toll on my relationship with her. We're still together, we're engaged, but because I felt gaslighted, I didn't want to talk to her about it every day.

After the investigation, Dr. Thompson finally came to me and said, "we need to speak, you have something you want to get off your chest." I told her that a big reason why sexual harassment happened was because of the favoritism that she displayed and how she constantly cultivated this environment where you have to fight to be seen as the best the whole team. She scoffed and rolled her eyes. She told me that the department was tired of me. I felt completely alone, and everyone fucking hates me.

After that meeting, I took 6 weeks off for mental health reasons. I switched labs and now I'm in another lab. I don't talk to Dr. Thompson anymore, but I still feel like I'm in survival mode. I've started to enjoy doing science again. but I wasn't good; like I was still crying a lot. It takes me an hour to do tasks that used to take me 5 minutes. I have intense flashbacks and I'm not able to sleep. It's been going on for about a month now. I'm just trying to live in the moment now – just trying to string days together like just taking it one day at a time.
