# Supplementary material for: Prevalence and Impact of Single-Day Events of Sexual Harassment, Racial Mistreatment, and Incivility on Biomedical Health Trainees: A Mixed-Methods Study
Source: Behav Sci (Basel). 2026 Mar 6;16(3):380. doi: 10.3390/bs16030380 (PMC13024630; doi:10.3390/bs16030380)
Supplement: Supplementary file 1 [file behavsci-16-00380-s001.zip › Supplementary Files/Study 2 Links to Diana and Sarah Videos.pdf]

24 Hour Negative Experiential Events Study – Follow up qualitative study (Study 2)

Links to Videos of “Diana” and “Sarah”

1. “Diana”: https://vimeo.com/686270245/c72025581f
2. “Sarah”: https://vimeo.com/686270571/538f9cf1a0
