# Supplementary material for: Prevalence and Impact of Single-Day Events of Sexual Harassment, Racial Mistreatment, and Incivility on Biomedical Health Trainees: A Mixed-Methods Study
Source: Behav Sci (Basel). 2026 Mar 6;16(3):380. doi: 10.3390/bs16030380 (PMC13024630; doi:10.3390/bs16030380)
Supplement: Supplementary file 1 [file behavsci-16-00380-s001.zip › Supplementary Files/Study 2 Sai Li Interview Transcript.pdf]

Sai Li Interview

Interviewer: Can you tell me a little bit about how all of this started and the nature of your relationship with Amit and how it all began?

Sai Li: Amit was a postdoc when I first joined the lab as a graduate student. Amit tried to help out at the beginning, but he's also, like, very sloppy with keeping things sterile. Like he would get ethidium bromide, which is this cancer-causing reagent all over the place. You're supposed to be careful with it. Jim, our mentor, will say that it's not that dangerous. Me on the other hand, I don't want to risk that for my offspring. So, a lot of us were women in the lab and we all felt similarly. He would also forget to turn off his flame. He would just leave his burner burning, walk away and we were all like, "hey you can't do that. Can you please pay attention to whether your flame is burning?" He was just like, "oh yea that's just me, ha ha ha, shucks." Jim, would be like, "well Amit means well," as in like, let's just give him some time.

One thing Amit would do that I would try to ignore, he would make comments 'cause he was very over the top, like that was part of his "humor," like "I would do anything for you," or "anything for a pretty girl like you." I just – just didn't react. I was a new student so I didn't want to rock the boat. I didn't want to really make a big deal out of it because he wasn't touching me; he wasn't like trying to coerce me on a date or something. He was married at the time. He wasn't doing anything past those comments and like I said I was a new student so I didn't wanna rock the boat. But, I wouldn't try to encourage the responses and I knew that if I tried to, like complain to him about it, he would just say, "oh why can't you take a compliment?"

Interviewer: How did that make you feel? What did you notice like, in your body, when he made those comments?

Sai Li: Physically I felt like I tensed up a bit just because I have been assaulted in the past. And then like emotionally I was just- it was maybe a little triggering. Emotionally I just put up this wall. Like a part of it was also just like "maybe I'm being too sensitive?". And people might say like "oh yea, India, generally patriarchal," and that might be the case but it would be unacceptable to talk about your wife the way he did and to compare her to other women the way he did. I've met plenty of Indian men who are complete gentlemen, so I don't consider that an acceptable excuse.

Interviewer: Okay, so how did things change with him once Jasleen joined the lab? What

started happening at that point?

Sai Li: Jasleen was placed under Amit's care as a rotation student. The relationship between Amit and Jasleen was cordial, at first. She had some pretty bad first and second rotation experiences, like, someone saying that she wasn't a serious scientist, like basically just insulting her, her work ethic, on a constant basis. Cause there's just toxic people everywhere. Amit would belittle her in front of Jim. She'd be like Amit called me- It was bad, it was so bad. She would come to me crying like three or so times a week.

Interviewer: So, multiple times a week, the things that Amit would say to her would just get to the point where she would break down and it would take multiple hours to kind of console her and get her to where she could go back to working with this person who is just going to say these horrible things again and you felt like you couldn't go to your mentor and actually get the support you needed in that situation.

Sai Li: I was trying to be as neutral as possible because, my mentor, Jim, doesn't like confrontation. I don't like confrontation either. But, I had to be the one to, like get Amit to calm down and try to, like, reason with him. I was like "Amit, you can't talk to women like that, you can't call them stupid and say that their mentor doesn't care about them." And he's like "oh no no, this is how we talk to women in my village all the time. She's from home, she's from the same cultural background so it feels like I I'm just talking to her the way I would with a person at home." And I'm like "mmmm no, no." And Jim was like, "well Amit is from a different cultural background. I was like, "Jim, this doesn't make any sense. He's here now, he did his PhD in the states, he did his masters in the UK. He's been in western society for at least 10 years. That's enough time for someone to pick up on the cues."

Jasleen will easily lose trust in Jim now. As soon as he says something a little off, it's almost like she goes a little bit back to the beginning. Amit would tell her, "Oh Jim doesn't care about you." Which is a lie. Jim never said that. It's just that Amit claimed that Jim said that. I was trying to explain this to Jim. I was like, "Jim, all of us are Asian. All of us have a strong sense of hierarchy." And he was like, "yea but we're here now." And I'm like well you can't have it both ways.

Interviewer: So when it finally all came to a head, tell me a little bit about how that all affected you. How it affected you emotionally. How it affected you physically. How it affected your job, like your ability to do your work.

Sai Li: : Physically, I lost 10 pounds which I could definitely not afford to lose. I weighed 116

pounds. I didn't even realize I was losing weight. That's how out of it I was. It was definitely affecting my physical and mental health too. Like when it got towards the end, I couldn't sleep with how angry I was. I feel like I was dealing with my own anxiety and stuff on top of Jasleen's being bullied and then like trying to make sure I didn't antagonize Amit too much because the more I antagonized- He wouldn't take it out on me, he'd take it out on Jasleen which was the crappy part of it too.

Interviewer: So it sounds like there was a lot going on. This feeling of anger that you had then it sounds like that was kind of the big persistent feeling that you had throughout this. A lot of anger and invalidation. What other feelings would you add into that?

Sai Li: Frustration, which to me is just a synonym for anger. Helplessness? Like I guess frustrated with myself because on some level I just wanted- I was putting so much effort into lab dynamics I just wanted the lab dynamic to be okay; but despite all of my efforts we were just like, scraping by. Cause Jasleen was still having her breakdowns. It was like I was gaslighting myself because it wasn't until I watched the 'Picture a Scientist' movie where I was just like, "hey I'm doing all the lab chores, if that's sexual harassment, I'm being sexually harassed." It also gave me something to point at. It gave me something to be like, "look! It's a real thing."

When it got to a breaking point right when Amit was leaving that's really when it was the worst. That's also when Jim really realized how bad the situation was because it took for all of us to be in tears. But instead of trying to talk Amit down, he just did Amit's chores for him.

Interviewer: You're still working on getting your doctorate. How has all of this influenced your thoughts post-graduation? Like continuing in academia, going into industry instead? How has this influenced your thoughts of what you want to do with your future?

Sai Li: It's influenced it a lot. Academia will lose me. It's not worth the existential uncertainty of an academic postdoc, no matter how interesting the science is. I don't wish it upon anyone else and I don't want to have to go through it again. Because if I join another lab and I end up in another situation like this where I'm being invalidated by my mentor, I'm keeping the peace and I'm like managing people's needs and emotions for them that they should definitely be able to take care of themselves. Growing up in science, like, as an intern and as an undergrad, a lot of the women who were my mentors, they would give me this advice and it felt like they were warning me of something terrible that was going to happen.
