# Supplementary material for: Prevalence and Impact of Single-Day Events of Sexual Harassment, Racial Mistreatment, and Incivility on Biomedical Health Trainees: A Mixed-Methods Study
Source: Behav Sci (Basel). 2026 Mar 6;16(3):380. doi: 10.3390/bs16030380 (PMC13024630; doi:10.3390/bs16030380)
Supplement: Supplementary file 1 [file behavsci-16-00380-s001.zip › Supplementary Files/Study 2 SH interview questions.pdf]

Sexual Harassment Survivor Interview:

Thank you for agreeing to this interview. My name is (identity withheld), and I am a doctoral candidate in the Clinical Psychology program here at IUPUI. I have completed several graduate courses in clinical psychology, and I have completed over 1400 hours of clinical training including over 400 hours as a therapist in different counseling programs or clinics in this area. My clinical supervisor and advisor is Dr. (identify withheld), who is a Professor of Psychology and a licensed psychologist.

I will be talking with you today about your experiences of sexual harassment. If you consent, our interview will be recorded (audiotaped), so that we can create a transcript of it. With us today is <name 1> who is an actor. <name 1> will be re-enacting this interview, which will be videotaped. The videotape will be edited, and you will have a chance to review it and give us your permission to use it for future training and research purposes. We will make sure that any identifying names or information that can identify you or your situation is changed to something fictitious.

Also with me today is <name 2>, who is a member of the research team. <name 2> can answer questions about the research that I may not be able to answer. <name 2> will also be taking notes to help the actor portray you in the videotape. Please know that both <name 1> and <name 2> and I will keep everything you tell us today confidential, to the degree the law allows. If you report any child abuse or intent to harm others or yourself, then we are required by law to report it to authorities.

You will be paid $200 for completing this interview in such a way that we can use a re-enactment of your video for training purposes. There will be no other compensation for this interview or for using the video in future training programs.

The purpose of the video and my interview will be to obtain a realistic depiction of how a person experiences sexual harassment and its aftermath. These interviews will be used to help trainees develop a true, empathic understanding of what it feels like to be sexually harassed. After viewing each video, trainees will engage in self-reflection exercises to develop their empathy and to develop a deeper commitment to making sure that they do not engage in sexual harassment and that they will stop others from engaging in sexual harassment. These videos will also be used in research to test their effectiveness.

Finally, although I am a clinician in training and I will be interviewing you as if you were talking to a clinician, I am not serving as your therapist for this interview. Furthermore, our interview does not obligate me to report your story to any authority here at IUPUI who would conduct an investigation. At the end of our interview, I will provide you with a list of resources you may contact if you wish to pursue an investigation, would like to speak to a therapist in their capacity as a therapist, or would like more information.

Do you agree to continue with this interview and to the conditions I have described? Do you have any questions.

First, I am going to ask you not use actual names. So let's decide what names you would like to use. For example, what is a pretend name I can call you, e.g., Susan, Rhonda, John. Second, what name or names would you like to use for the person or people who sexually harassed you. If it was a professor, perhaps, we could say Dr. Jones or Dr. Lincoln – What name would you like to use? (X) Was there another person involved? If so, what name would you like to call them by? (Y) Finally, will you be mentioning any other

people, perhaps someone you confided in, or someone who may have known what was going on. What names should we use? (Z)

Begin taping

1. Can you tell me how this started? Describe the nature of your relationship with X in the beginning. How did you come to know X.?
2. Please describe how the situation with X changed or continued over time. Describe how the harassment persisted.
3. Describe what you were feeling and what you were thinking as these events were happening.
4. Describe what you did or did not do while this was happening.
5. Tell me how you were feeling or thinking about yourself while this was happening.
6. Tell me about how this experience (these experiences) affected you
   a. For example, how did it affect you emotionally or physically?
   b. Describe what effects it had on your studies or job?
   c. What effects did it have on your relationships with other people?
   d. How did the experience affect how you think about yourself?
7. If you confided in a friend of family member about what happened or was happening to you, describe how they reacted and what they did or did not do to help you. Again, please use fictitious names.
8. Did you report your experience to anyone you thought may be able to do something about it? If so, please describe what happened?
9. Is there anything else you would like to tell me about this experience?

Thank you very much for this interview. I know it wasn't easy and it is painful to relive these experiences. Hopefully with your help we can create effective training programs and other interventions that will help make sure this doesn't happen to other people.

Now that we have completed the interview, do you still agree to let us either use a re-enactment of this interview for training and research purposes? One of the researchers will contact you and let you see the re-enacted version so that you can give or retract your permission to let them use the video for training or research.

I am going to give you a list of resources you can contact if you would like to additional help or support with this situation.

Thank you
